# Metabolic interactions affect the biomass of synthetic bacterial biofilm communities

**DOI:** 10.1128/msystems.01045-23

**Published:** 2023-11-16

**Authors:** Xinli Sun, Jiyu Xie, Daoyue Zheng, Riyan Xia, Wei Wang, Weibing Xun, Qiwei Huang, Ruifu Zhang, Ákos T. Kovács, Zhihui Xu, Qirong Shen

**Affiliations:** 1 Key lab of organic-based fertilizers of China and Jiangsu provincial key lab for solid organic waste utilization, Nanjing Agricultural University, Nanjing, Jiangsu, China; 2 Bacterial Interactions and Evolution Group, DTU Bioengineering, Technical University of Denmark, Kongens Lyngby, Denmark; 3 Institute of Biology Leiden, Leiden University, Leiden, the Netherlands; University of California, San Diego, La Jolla, California, USA

**Keywords:** soil microbiology, SynComs, biofilms, network analysis, metabolic modeling

## Abstract

Microbes typically reside in communities containing multiple species, whose interactions have considerable impacts on the robustness and functionality of such communities. To manage microbial communities, it is essential to understand the factors driving their assemblage and maintenance. Even though the community composition could be easily assessed, interspecies interactions during community establishment remain poorly understood. Here, we combined co-occurrence network analysis with quantitative PCR to examine the importance of each species within synthetic communities (SynComs) of pellicle biofilms. Genome-scale metabolic models and *in vitro* experiments indicated that the biomass of SynComs was primarily affected by keystone species that are acting either as metabolic facilitators or as competitors. Our study sets an example of how to construct a SynCom and investigate interspecies interactions.

**IMPORTANCE:**

Co-occurrence network analysis is an effective tool for predicting complex networks of microbial interactions in the natural environment. Using isolates from a rhizosphere, we constructed multi-species biofilm communities and investigated co-occurrence patterns between microbial species in genome-scale metabolic models and in vitro experiments. According to our results, metabolic exchanges and resource competition may partially explain the co-occurrence network analysis results found in synthetic bacterial biofilm communities.

## INTRODUCTION

Despite a wealth of information available on the microbiota composition gathered from sequencing techniques, little is known about the interspecies interactions that govern their assemblage and dynamics. Yet, the size and complexity of natural microbial communities are often too large to be manipulated. Synthetic microbial communities (SynComs) have been proposed as model systems to overcome these challenges (1). Several SynComs with moderate complexity and high controllability have been developed to represent different natural environments, such as plant rhizosphere (2–6), phyllosphere (7, 8), and intestine (9–11). In nature, most bacteria aggregate as sessile multicellular communities that are embedded in the self-produced extracellular matrix called biofilm (12, 13). Biofilm communities have emergent properties that are not predictable from free-living bacterial cells (14), including enhanced biomass production (15), stress tolerance (16, 17), and improved resource acquisition (18). The spatial organization of biofilms facilitates the intermixing of cooperating species and the spatial segregation of competing species (19). Understanding how bacterial interactions affect the assemblage of bacterial communities has direct applications in biotechnology, agriculture, and health (20–24).

Studies on SynComs reported that the assemblage and robustness of communities can be affected by several factors, including pH (10), temperature (25), spatial distribution (26), initial abundance (27), niche specificity (28), nutrient availability (29), and keystone species (4). Five principles need to be considered when developing SynComs: representativity, stability, reduced size, accessibility, and tractability (30). To assemble a representative and stable SynCom, microbial co-occurrence network analysis could serve as guidance for the selection of isolates (31). Positive or negative associations indicate candidate taxa. This method has been applied to investigate factors affecting host microbiome variation (32), link taxa to biological functions of interest (33), identify potential biotic interactions (34), and explore habitat differentiation (35). However, the ecological relevance of predicted interactions remains poorly understood (36). The connectedness and strength of positive or negative interactions are not experimentally verified. Another emerging method for exploring microbial interactions is genome-scale metabolic modeling, which can provide insights into metabolic interaction potential and metabolic resource overlap in multi-species communities (37, 38). Accessibility and tractability require a community to contain limited members, and the community members are cultivable and can be promptly and accurately quantified. This can be achieved by colony-forming unit (CFU) counting (4, 39), fluorescent labeling (40), and quantitative PCR (15). While CFU counting is simple to employ but time-consuming, the latter two approaches are more efficient but require extensive initial preparation time and effort. Reduced size permits the manipulability of a SynCom.

In this study, we constructed multi-species biofilm communities using isolates from a rhizosphere and predicted their interactions by analyzing co-occurrence networks. We evaluated the role each member played in community biomass and its impact on the other members of the community. The potential metabolic interactions were further investigated through experiments and metabolic modeling. Our results suggested that microbial interactions could be explained by metabolic exchanges or resource competition. We propose that our study could inform the rational design of synthetic communities.

## RESULTS

### Source of SynCom members

As co-existence facilitates biofilm formation in multi-species communities (41), we adopted co-existence as the first criterion of SynCom selection. To assemble a soil biofilm community, we co-cultured two different rhizosphere soils with *Bacillus velezensis* SQR9 (a strong biofilm previously widely studied in our laboratory, abbreviated as Bac) to form pellicle biofilms (Fig. S1A). After 24 h of incubation, we determined the bacterial composition of the biofilm and the solution underneath using the 16S rDNA amplicon sequencing method. As a result, 14 genera and 2 families co-existed in the culture condition (Fig. S1B). From our bacterial collection (42), we selected 11 isolates that corresponded with the observed co-existing taxa to represent the soil biofilm community (Fig. S2). These isolates are derived from three different phyla: Firmicutes, Proteobacteria, and Bacteroidetes.

**FIG 1:**
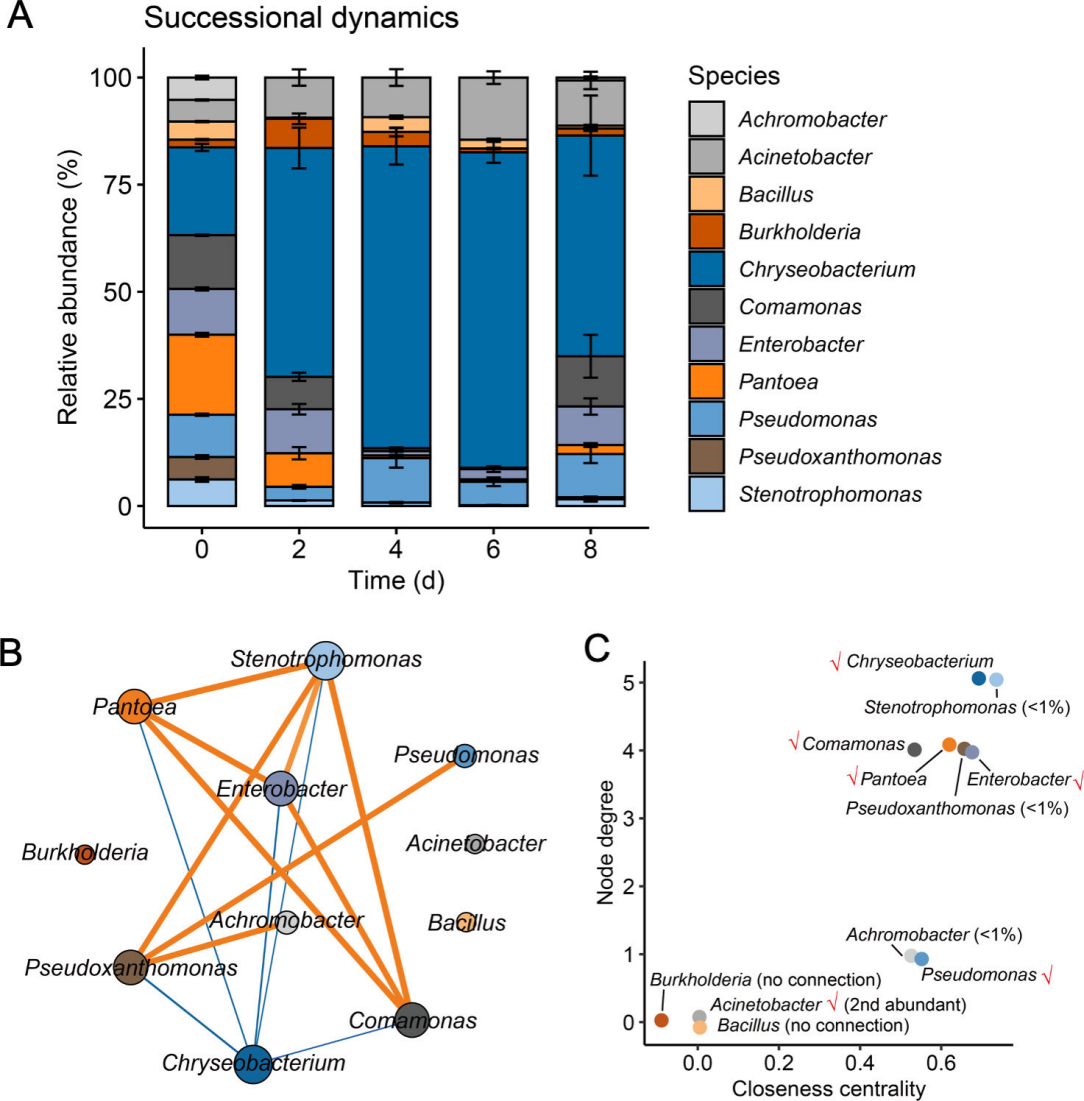
Dynamic changes in the initial 11-species consortium composition in the biofilm. (**A**) The relative abundance of isolates determined by the 16S rRNA gene amplicon sequencing method. Data presented are the mean ± SD. *n* = 8. (**B**) Co-occurrence network of bacteria within biofilm communities. Each node represents a bacterial species; node size is proportional to node degree. Line width indicates the interactive strength of interaction, and color indicates the sign of correlation (orange indicates positive, blue indicates negative). A positive correlation means that as one isolate increases, the other isolate also tends to increase, and vice versa. (**C**) Selection of six isolates. Node degree is the number of direct correlations to a node in the network. Closeness centrality indicates the average distance from a given starting node to all other nodes in the network. Ticks indicate isolates selected in the following reduced community. These isolates were selected based on either high abundance or high correlations, as indicated by node degree and closeness centrality.

Generally, biofilm development undergoes three major events: aggregation, growth, and disaggregation (43). To evaluate the stability of the SynCom, we expanded the volume of cultivation to 400 mL and tracked the dynamic changes in bacterial composition across biofilm stages (Fig. S3A): aggregation (2 days), growth (4 days), maturation (6 days), and disaggregation (8 days). The bacterial composition stabilized at the growth and maturation stages. Chr (*Chryseobacterium rhizoplane*) was identified as the most predominant member, followed by Aci (*Acinetobacter baumannii*) as the second most abundant species throughout the biofilm formation process (Fig. 1A). Three isolates, Ach (*Achromobacter denitrificans*), Pxa (*Pseudoxanthomonas japonensis*), and Ste (*Stenotrophomonas maltophilia*), rapidly declined and could not establish themselves during biofilm growth (4 and 6 days). At the biofilm disassembly stage (8 days), the sticky, robust biofilm has dispersed as small, fragile aggregates. Most cells have lysed, and the relative abundance of rare isolates has increased. Growth rates and growing capacity are not sufficient to explain the differences observed in the relative abundance composition of isolates (Fig. S4). We hypothesized that microbial interactions could contribute to the compositional changes. Therefore, network co-occurrence analysis was employed to infer the correlations (Spearman’s correlation coefficient *r* > 0.6, *P* < 0.01) among the species during biofilm formation (Fig. 1B). The co-occurrence network revealed 9/14 positive correlations and 5/14 negative correlations. Intriguingly, the negative correlations all involve Chr, suggesting that the increment of Chr is correlated with the reduction of other isolates. Aci, Bac, and Bur (*Burkholderia contaminans*) had no connection with others, indicating that they had little influence on the growth of other isolates (Fig. 1B). The connectedness of isolates was further evaluated by node degree and closeness centrality (Fig. 1C). Node degree is the number of edges the node has, that is, the number of other community members a strain is significantly correlated to. The higher the degree is, the more central the strain is. High closeness centrality indicates that the node is closely connected to other nodes and is central in the network. As a result, six isolates—Chr, Ste, Com (*Comamonas odontotermitis*), Pan, Ent (*Enterobacter bugandensis*), and Pxa—are closely connected with other isolates and are central to the network. Ach and Pse are only connected with Pxa. Other nodes had no connection to the network.

In the subsequent experiments, we selected six isolates to examine bacterial interactions. They were selected based on high relative abundance (Chr, Aci, Pse) at biofilm growth and maturation stages (4 and 6 days) or high closeness centrality (Pan, Com, Ent). Chr fulfilled both criteria and was hypothesized as the keystone negative species in the reduced SynCom. Other species were excluded either due to their extremely low abundance at the biofilm maturation stage (Ach, Pxa, Ste) or due to the lack of correlations with the other species (Bac, Bur).

### The biomass of multi-species biofilm is affected by central nodes

The co-occurrence network suggested that Pan, Com, and Ent are positively correlated with each other, while they are all negatively correlated with Chr. Aci and Pse are not connected with the selected nodes. We hypothesized that the removal of central nodes (Pan, Com, Ent, Chr) would have a significant impact on community composition and biomass, while the removal of non-connected nodes (Aci, Pse) would have no impact. To evaluate the importance of each isolate on the community, we applied the “removal” strategy: the “full” community consists of six isolates, and then one isolate was dropped out to obtain the five-species reduced communities, abbreviated as “Rm” communities. The role of each isolate is represented as changes in community biomass by removing a certain isolate from the full community.

**FIG 2:**
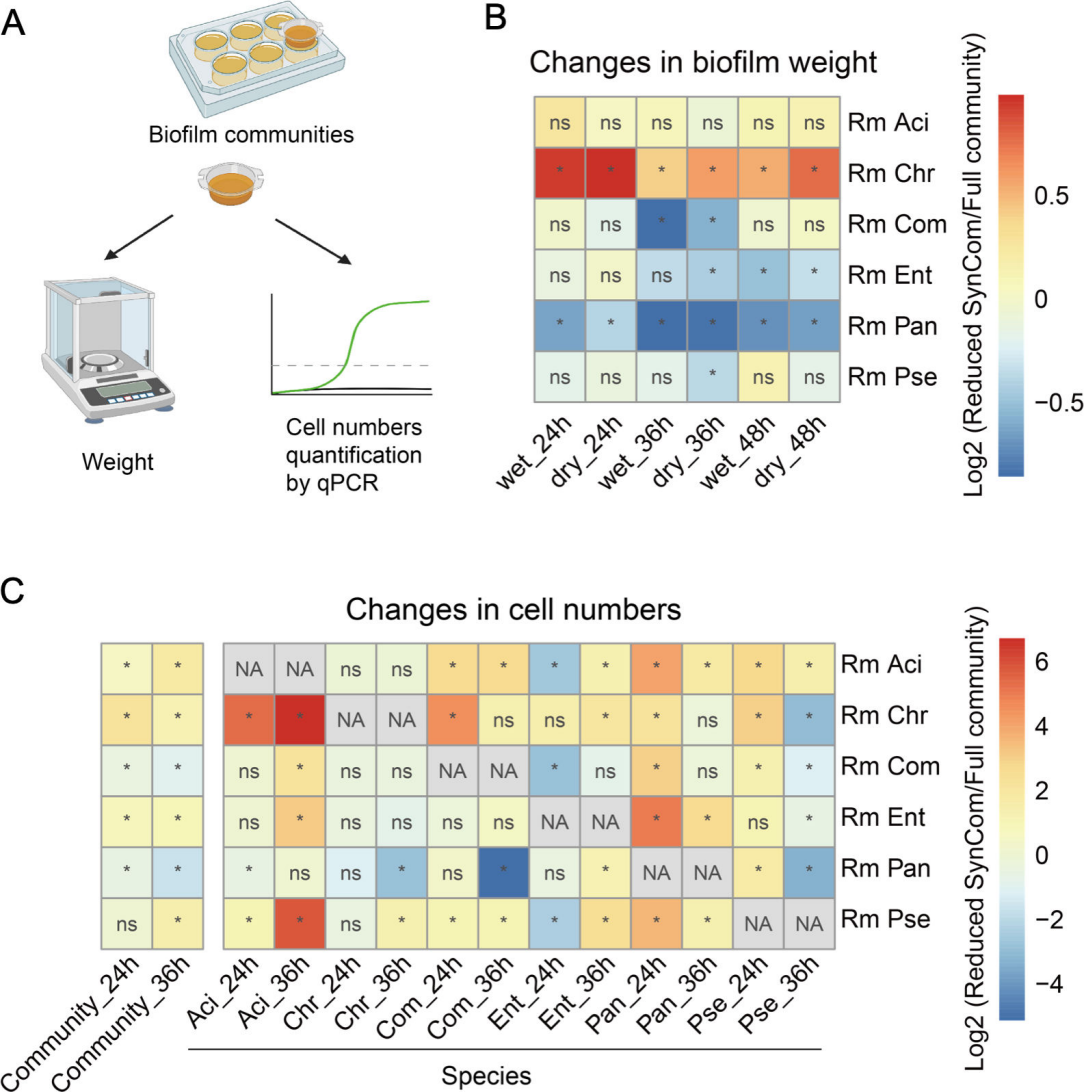
Productivity of the reduced SynComs. (**A**) Experimental design. (**B**) Changes in biofilm weight and population cell numbers. “*” indicates that the value of the reduced community is significantly changed compared with the full community,“ns” indicates no significance (Welch’s ANOVA, Games-Howell post hoc test, *P* < 0.05, *n* = 6). The values of the replicates are shown in Fig. S4. (**C**) Influence of removing one isolate on SynCom composition. “*” indicates that the cell numbers of the isolate in the reduced community are significantly different compared with those in the full community, “ns” indicates no significance, “NA” indicates no data (Welch’s ANOVA and Games-Howell post-hoc test, *P* < 0.05, *n* = 6). The values of the replicates are shown in Fig. S5. Rm Aci, Rm Chr, Rm Com, Rm Ent, Rm Pan, and Rm Pse represent the five-species communities resulting from the removal of *Acinetobacter baumannii* XL380, *Chryseobacterium rhizoplanae* XL97, *Comamonas odontotermitis* WLL, *Enterobacter bugandensis* XL95, *Pantoea eucrina* XL123, and *Pseudomonas stutzeri* XL272, respectively.

Biofilm biomass was first assessed by weight (Fig. 2A and B). We minimized the volume of biofilm cultivation to 10 mL to allow massive parallel sampling of the different combinations. Importantly, the smaller volume and reduced bacterial diversity change the biofilm phenotype and development. The biofilm started to disperse at 36 h (Fig. S3B); thus, three time points were tested: 24, 36, and 48 h (Fig. 2B; Fig. S4). “Rm Chr” resulted in higher biofilm wet weight and dry weight at all three time points (Welch’s analysis of variance [ANOVA, Games-Howell test, *P* < 0.05). Conversely, “Rm Pan” led to lower biofilm weight at all three time points (Welch’s ANOVA, Games-Howell test, *P* < 0.05). Removal of the other two positive nodes (Com and Ent) caused a reduction in biofilm weight at some time points. Removal of non-connected nodes (Aci and Pse) had little impact on community weight, which supports our hypothesis.

Next, we assessed community biomass by quantifying cell numbers in the biofilms. We compared the cell numbers in the reduced SynComs with those in the full community at 24 and 36 h to evaluate the impact of removing one isolate on community composition (Fig. 2C; Fig. S6). The cell numbers were quantified by quantitative PCR (qPCR) using strain-specific primers (Data S1; Table S1). In consistency with weight quantification, Rm Chr significantly increased community cell numbers at both time points, while “Rm Com” and Rm Pan reduced community cell numbers at both time points (Welch’s ANOVA, Games-Howell test, *P* < 0.05). Interestingly, Rm Chr increased the cell numbers of each isolate at 24 h, which confirmed its negative role as observed in network analysis. Removal of Com caused a reduction in cell numbers of Ent, which confirmed their positive correlation as observed in the network analysis. Rm Pan resulted in the reduction of Chr, Com, and Pse at 36 h, suggesting that Pan was important for supporting their growth at the biofilm maturation stage. The contradictory effect of “Rm Ent” on weight and cell numbers made it difficult to assign a clear role. Removal of Aci and Pse also increased the cell numbers of several isolates, which was not consistent with the no correlation observation in the co-occurrence network.

Taken together, we proposed Chr as a keystone negative species in the SynCom in terms of negatively affecting community weight and inhibiting the growth of each isolate. Pan is predicted to be a positive keystone species because its removal decreased the growth of three members and decreased the overall community biomass.

### Metabolic facilitation can explain the positive interaction

**FIG 3:**
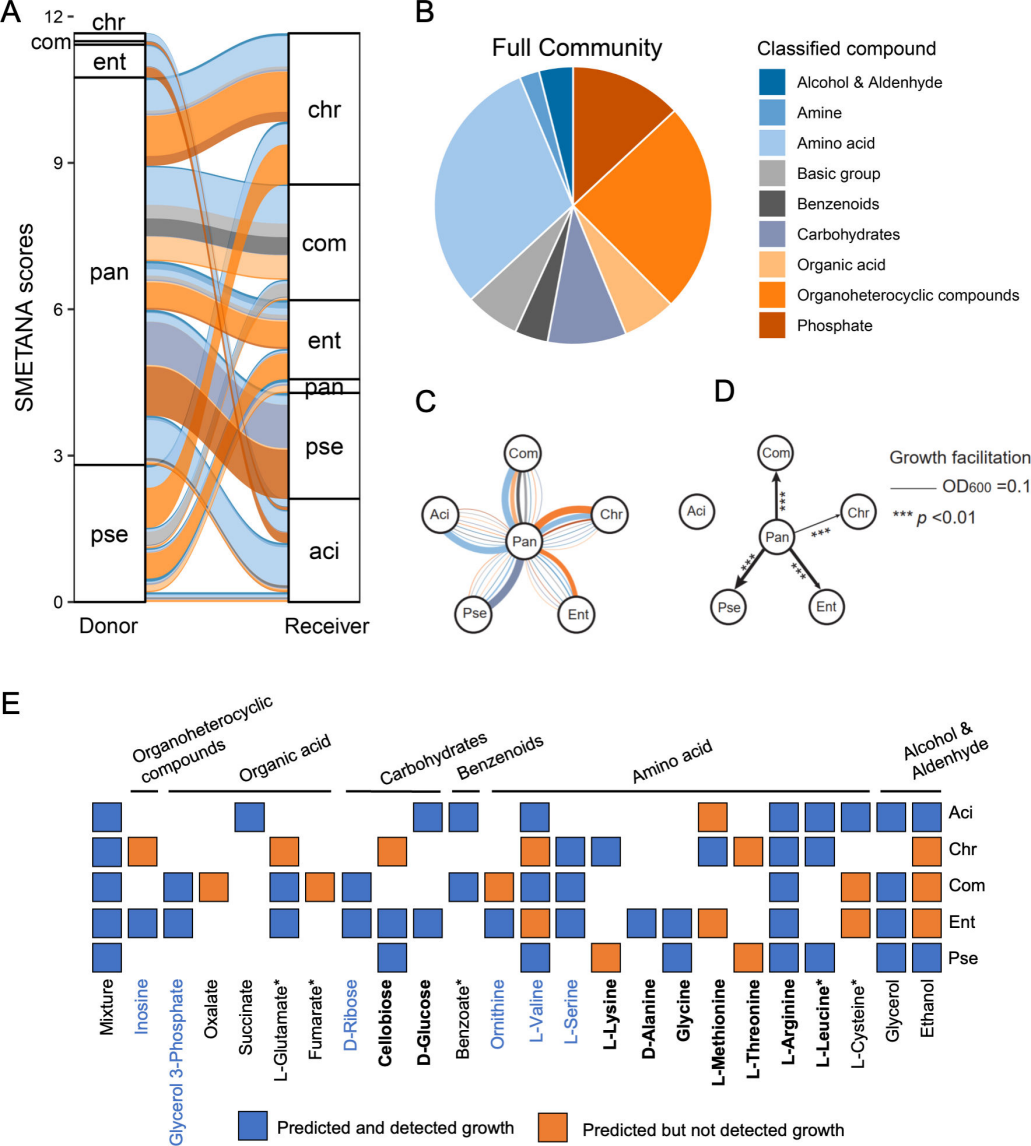
Metabolic facilitation. (**A**) Alluvial diagram showing the metabolic interaction potential of the full community simulated by genome-scale metabolic modeling. SMETANA scores are assigned to strip thickness. (**B**) Pie chart showing the proportion of compounds exchanged within the metabolic model-simulated community. (**C**) A flower plot showing metabolic interactions involving Pan (centered in each panel) as a donor. The thickness of lines is proportional to the magnitude of the SMETANA score. The colors of all plots are the classification of compounds. (**D**) Growth facilitation is assessed by growth in the spent medium. The spent medium was prepared by growing the corresponding species in M9 medium with 0.2% glucose until the glucose was detected, then filter sterilized. The species were grown in the filtered spent medium for 24 h, and the growth was measured as the optical density of 600 nm (OD_600_). Line width indicates the growth facilitation, that is the final OD_600_ substact the initial OD_600_. Only growth facilitation larger than 0.1 was shown in the figure. The values were compared with those of no inoculation control. ***P < 0.01, *t* test. (**E**) Growth of isolates in predicted Pan metabolites. Growth was tested by measuring OD_600_ in M9 medium (black text), by phenotype microarray assay (blue text), or by both methods (black bold text). OD_600_ values higher than 0.1 or omnilog values higher than 20 were defined as growth. Based on the tested compounds, the accuracy of predicted metabolite exchange is 69.5% (41/59). Accuracy is calculated as the number of “predicted and detected growth” divided by the number of carbon sources tested. The mixture was not considered in the calculation. See Fig. S8 for more details.

To gain insights into the metabolic interaction potential of the SynCom members, genome-scale metabolic models were established using the CarveMe pipeline. The M9 glucose minimal medium was set as the input medium because this medium can be experimentally validated. We derived the likely exchanged metabolites across communities and the strength of metabolic coupling from the SMETANA score (37). In the full community, Pan served as the principal metabolic donor, and all the other isolates received benefits from it (Fig. 3A). On the contrary, Chr acted as the highest metabolic receiver, while Pan had the least potential to be the metabolic receiver. Amino acids, organoheterocyclic compounds, and phosphate were the major exchanged metabolites (Fig. 3B). Aci and Com mainly receive amino acids from Pan; Chr receives organoheterocyclic compounds and amino acids from Pan; Ent mainly receives organoheterocyclic compounds from Pan; and Pse receives carbohydrates from Pan (Fig. 3C). Further experimental validation is necessary for these predicted exchanged compounds.

**FIG 4:**
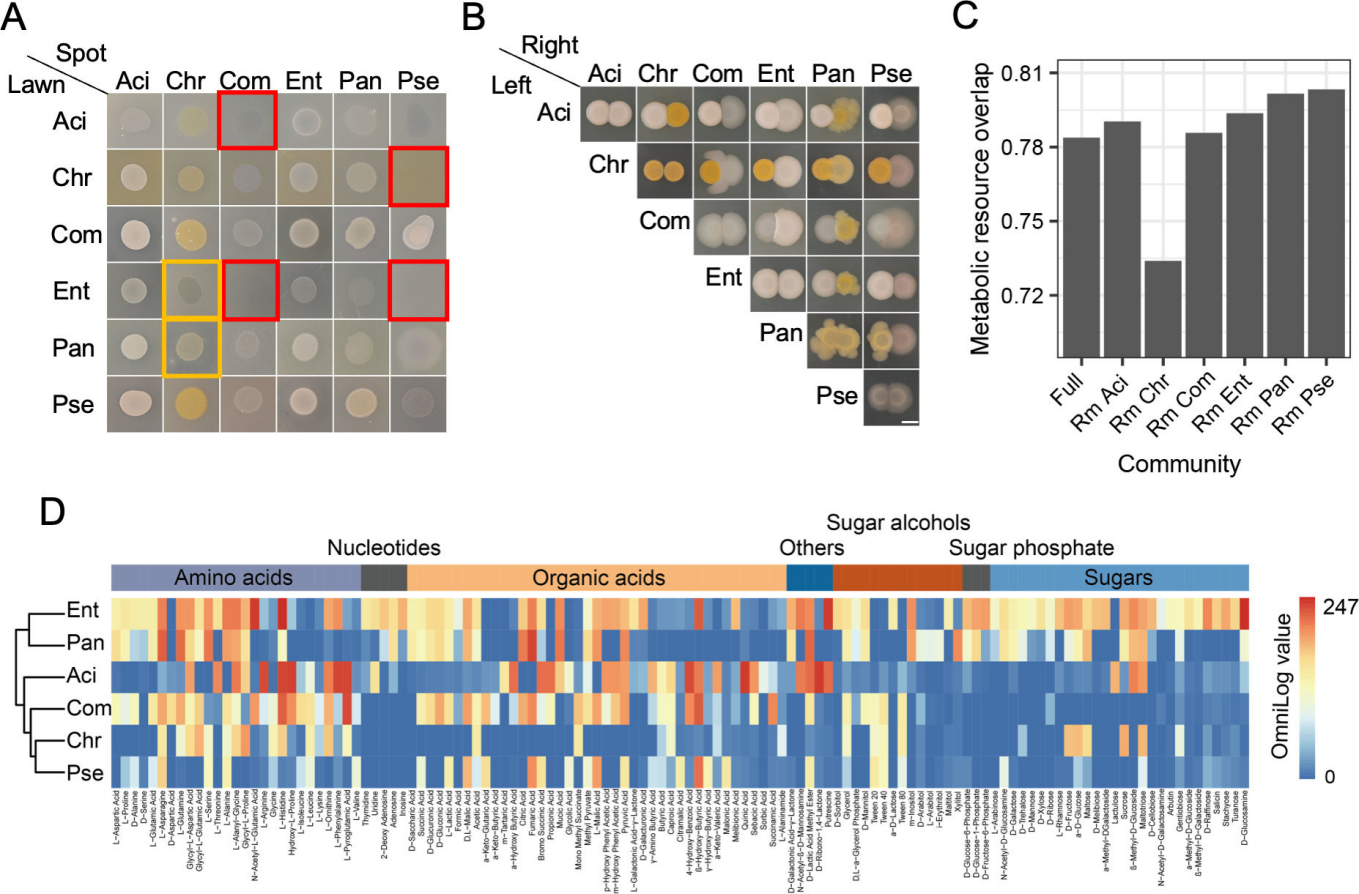
Resource competition. (**A**) Pair-wise spot assays. Five microliters of the two species was plotted next to each other at an optical density of 600 nm of 1. The scale bar represents 3 mm. Photos were taken after 48 h of incubation at 30°C. (**B**) A bar chart showing the metabolic resource overlap simulated by the metabolic modeling. (**C**) Spot-on-lawn phenotype. See Fig. S8 for details. (**D**) Carbon source metabolic ability measured by phenotype microarrays. Dye reduction signals by cells were automatically collected by the OmniLog instrument. The endpoint OmniLog values in test wells, subtracting the values in the blank control well, are shown. The OmniLog value indicates the cell respiration intensity.

Growth assays were used to determine the accuracy of the predicted metabolite exchange. First, cross-feeding was tested by measuring growth in spent media. Only four isolates were able to grow individually in the M9 medium with 0.2% glucose as sole carbon: Ent and Pse displayed high growing capacity, Aci and Pan grew moderately, and Chr and Com showed no growth (Fig. S7A and B). Therefore, Ent, Pse, Aci, and Pan could have the potential to serve as metabolic donors in the M9 glucose medium. Each isolate was cultured in the M9 glucose medium until glucose was under the limit of detection. The obtained spent medium was then filter-sterilized and used to cultivate every other member of the community and themselves (Fig. 3D). The spent medium of Pan could support the growth of Aci, Chr, Ent, and Pse (Fig. S7C through F). On the contrary, no isolates could grow in the metabolic by-products of Aci, Ent, or Pse. Second, the abilities of each isolate to grow upon the predicted metabolites from Pan were tested using growth assays in M9 medium (Fig. S8) or phenotype microarray assays (Fig. 4D). Based on the tested metabolites, the accuracy of predicted metabolite exchange is 69.5% (Fig. 3E). These results might explain the positive role of Pan in the biofilm community as a metabolic donor.

### Resource competition can explain the negative interaction

To test the possibility of direct antagonism, we performed spot-on-lawn and pair-wise spot assays on tryptic soy broth (TSB) agar plates (Fig. 4A and B). Both Aci and Ent completely inhibited the growth of Com. Similarly, Chr and Ent inhibited Pse growth. However, these inhibitions were not caused by direct antagonism, as no inhibition zones were observed in the respective spot-on-lawn assay plates (Fig. 4A, red square). In addition, no clear boundary was observed between the two strains when they were spotted adjacent to one another (Fig. 4B). Therefore, the negative influence of Chr on the other SynCom members is not due to interference or competition. Interestingly, Pan and Ent could change the colony color of Chr from light yellow to white (Fig. 4A, yellow square), indicating an altered secondary metabolism in Chr.

Negative interspecies interactions in the SynComs might be explained by exploitation competition. Using metabolic modeling, the metabolic resource overlap was simulated (Fig. 4C). We observed high metabolic resource overlap across all communities, indicating intense resource competition among SynCom members. The removal of Chr minimized the metabolic resource overlap, while the removal of each isolate resulted in higher resource competition. Chr may act as a strong competitor for other strains based on metabolic modeling, which requires further validation. We tested the growth of other members in the supernatant of Chr to gain insight into this hypothesis. Results showed that Chr consumed the majority of nutrients in TSB after 24 and 36 h, thereby greatly reducing the growing capacity of the other strains (Fig. S10). The extent of resource competition needs to be further explored by experiments.

We assessed the carbon source metabolic ability by high-throughput phenotypic microarrays (Fig. 4D). Ent and Pan were generalists that could utilize a wide variety of carbon sources. Resource competition caused by similar nutrient preferences could explain the negative correlation between Ent and Pan as observed in the reduced SynComs. Other community members specialized in using amino acids and organic acids as carbon sources while having a limited ability to utilize nucleotides, sugar alcohols, and sugars (Fig. 4D). Therefore, we propose that Chr might inhibit the growth of other community members by rapidly depleting nutrients rather than through direct antagonism or occupying broad metabolic niches.

## DISCUSSION

A key concern in SynCom research is to understand how microbial interactions affect community composition and biomass. In this study, we evaluated the bacterial interactions by co-occurrence network analysis and tested the contribution of each individual to community biomass by the removal strategy. Our SynCom system identified *Pan* (*P. eucrina*) and *Chr* (*C. rhizoplanae*) as important drivers of community interaction networks through metabolic cross-feeding and resource competition.

Manipulating microbial consortia has a variety of applications; however, attempts to engineer them often fail to achieve the expected results owing to the unexpected effects of interactions within the community. To assemble a stable community, three main criteria should be considered: natural co-occurrence, tractability, and stability. First, to represent a natural co-occurrence, the community should contain members that also co-exist in natural settings. Natural co-occurrence ensures a higher possibility of stable co-existence as well as higher natural relevance than artificial combinations of isolates with unrelated origins. The model maize community (4), SXMP soil community (15), THOR community (44), metal working fluids community (39), cheese rind community (45), and Yeast-LAB community (46) were all constructed based on this criterion. Multiple species co-occurring in the natural microbiome could be pooled together to form an initial community. We assembled the initial community based on soil co-occurrence and co-existence in a laboratory biofilm. The second criterion is tractability. All the community members should be easily tractable by colony counting or qPCR, and the community should have an easily tractable trait, such as biofilm biomass. The third premise is a reduced community size. Several rules could be adopted to reduce community size, such as removing species that cannot establish themselves in the initial community (this study) or species that share similar genetic backgrounds (5). In this study, the community composition was tested by 16S rDNA gene amplicon sequencing and strain-specific qPCR methods. The community was minimized by network analysis; six correlated or abundant isolates were selected from the initial community. The stability of a reduced community and the ability to maintain all the isolates should be tested again at select time points of assembly.

We tested the positive or negative roles of the isolates as observed by network analysis through the so-called removal strategy (where certain members were removed from the SynCom). Only four of the six isolates (Chr, Com, Pan, and Pse) exhibited an expected influence on community biomass. Therefore, although a co-occurrence network could serve as the first step for predicting bacterial interactions, this approach is insufficient for conclusively identifying keystone species and requires experimental validation (8, 36). The removal strategy could be used to verify the role of the keystone species (4, 8).

After these three steps, further properties can be assessed according to the research interest, e.g., plant-growth-promoting properties, toxic fluid degrading ability, and stress resistance (4, 16, 39, 47). Furthermore, potential interactions can be detected using spent medium growth assays, pair-wise growth experiments, and whole-genome metabolic modeling. Resource competition plays a major role in shaping bacterial communities, while metabolic exchanges promote group survival (37, 48). In the present study, we simulated the metabolic interactions using metabolic models and assessed their predictability using phenotypic assays. Both the metabolic model and the experiments suggested that the positive keystone species, Pan, serves as a metabolic donor and supports the growth of other community members. On the contrary, the negative keystone species, Chr, might negatively affect community biomass through metabolic competition. It is predicted to be the largest metabolic receiver in the community by metabolic models. Removal of this isolate was predicted to decrease the metabolic resource overlap within the community. It grew rapidly and accumulated a high growing capacity, thereby decreasing the available nutrients for other strains. These results indicate that metabolic interactions play a key role in determining the composition of communities, which is also observed in the maize model community (49). Importantly, the accuracy of predicted metabolite exchange is 69.5% based on the tested compounds. This result could be attributed to the inherent uncertainty in genome-scale metabolic model reconstruction, such as the limited accuracy of genome annotation, the limitation of defined media, the lack of direct experimental measurements for most organisms, and the problems in network gap-filling (50). Alternatively, certain metabolic interactions that only occur in community contexts cannot be validated using currently available methods. Furthermore, certain substances are poorly soluble, making it difficult for them to serve as the sole carbon source for microbial growth. A more accurate estimation of community metabolic interactions can be achieved with the development of metabolic modeling methods and new approaches for detecting metabolite exchanges.

One technical limitation of our study is the use of different media for the biofilm experiments and the metabolic assays. No biofilm is formed by these SynCom members in a minimal medium, while the metabolite exchange cannot be easily tested in a rich medium, like TSB. Nevertheless, the observed cross-feeding in the minimal medium is likely to occur in the biofilm, as reported in a dual-species biofilm community (51). Furthermore, other mechanisms, such as nutrient availability, niche partitioning created by spatial and temporal heterogeneity, and trade-offs between nutrient acquisition and environmental tolerance, can promote coexistence between species (8, 28, 52, 53). Various properties and mechanisms of bacterial interactions can be derived from the studies using model communities. For example, studies on the Yeast-LAB community revealed that cross-feeding of amino acids might explain the co-existence of yeast and lactic acid bacteria in a variety of naturally fermented foods and beverages (46). Research on an SXMP soil community proposed the emergence of community intrinsic properties, such as enhanced biofilm formation and protection against grazing (15, 54). Furthermore, a three-species biofilm community displayed enhanced resistance to antibiotics and sodium dodecyl sulfate (SDS) (16).

Although the potential practical application of the here-developed SynCom is unclear, our research provides insights into how metabolic competition and cooperation simultaneously shape the community composition. The methodology of network co-occurrence analysis combined with qPCR quantification, metabolic modeling, and pair-wise interactions can be applied in diverse SynComs studies. Ultimately, such studies should translate to a deeper understanding of how microbial communities behave in their native environments, and this knowledge may be applied to wastewater treatment, disease suppression, and crop yield enhancement.

## MATERIALS AND METHODS

### Strains and genome sequencing

As shown in Table 1, 11 bacterial isolates derived from the cucumber rhizosphere (42) were selected for this study. These isolates were selected based on their co-existence in biofilms (see Results). The starting inoculum was prepared by growing overnight culture in TSB medium, centrifuged (5,000 × *g*, 2 min), and resuspended in 0.9% NaCl solution to an optical density of 600 nm of 1 (OD_600_, ~1). Multi-species starting inoculum was prepared by mixing equal volumes of each single species containing inoculum.

**TABLE 1:** Strains used in this study

Strain	Abbreviation
*Achromobacter denitrificans* XL100	Ach
*Acinetobacter baumannii* XL380	Aci
*Bacillus velezensis* SQR9	Bac
*Burkholderia contaminans* XL73	Bur
*Chryseobacterium rhizoplanae* XL97	Chr
*Comamonas odontotermitis* WLL	Com
*Enterobacter bugandensis* XL95	Ent
*Pantoea eucrina* XL123	Pan
*Pseudomonas stutzeri* XL272	Pse
*Pseudoxanthomonas japonensis* XL7	Pxa
*Stenotrophomonas maltophilia* XL133	Ste

The whole genomes of the selected isolates were sequenced by different companies at different times. The genomes of Aci, Com, Pxa, and Ste were sequenced using a combination of PacBio RS II and Illumina HiSeq 4000 sequencing platforms. The genomes of Aci, Pxa, and Ste were sequenced at the Beijing Genomics Institute (Shenzhen, China). Four SMRT cells Zero-Mode Waveguide arrays of sequencing were used by the PacBio platform to generate the subreads set. PacBio subreads (length < 1 kb) were removed. The program Pbdagcon (https://github.com/jgurtowski/pbdagcon_python) was used for self-correction. Draft genomic unitigs, which are uncontested groups of fragments, were assembled using the Celera Assembler against a high-quality corrected circular consensus sequence subreads set. To improve the accuracy of the genome sequences, GATK (https://www.broadinstitute.org/gatk/) and SOAP tool packages (SOAP2, SOAPsnp, SOAPindel) were used to make single-base corrections. To trace the presence of any plasmid, the filtered Illumina reads were mapped using SOAP to the bacterial plasmid database (http://www.ebi.ac.uk/genomes/plasmid.html, last accessed 8 July 2016). Raw sequencing data and the assembled genome have been deposited with the National Center for Biotechnology Information (NCBI) under the BioProject accession numbers PRJNA593376, PRJNA762936, and PRJNA762715. The genome of Com was sequenced at Majorbio Bio-Pharm Technology Co., Ltd. Raw sequencing data and the assembled genome have been deposited with the NCBI under the BioProject accession number PRJNA762695.

The genomes of Bur, Chr, Ent, and Pan were sequenced using the PacBio Sequel platform and Illumina NovaSeq PE150 at the Beijing Novogene Bioinformatics Technology Co., Ltd. Raw sequencing data and the assembled genomes have been deposited with the NCBI under the BioProject accession numbers PRJNA593683, PRJNA721858, PRJNA761942, and PRJNA762676. Pse was sequenced by reference 42. Genomes were automatically annotated by NCBI PGAP.

### Cultivation of soil community

To create a multi-species biofilm community, the co-existence criterion was adopted. In parallel with our previous study (42), the rhizosphere soil of cucumber was collected. Two types of soil were chosen: black soil and paddy soil. Bacterial abundance in the rhizosphere soil was roughly estimated by CFU counting on TSB agar plates. Equal volumes of soil suspension were mixed with *B. velezensis* SQR9 (OD_600_, ~1) and inoculated in 2 mL of TSB liquid medium at a 1:100 ratio. Biofilms formed at the air-liquid interface and the solution underneath were collected separately after 24 h of incubation at 30°C. Each treatment had three biological replicates. The genomic DNA of the samples was extracted using an E.Z.N.A. Bacterial DNA Kit (Omega Bio-tek, Inc.) following the manufacturer’s instructions. Universal primers targeting the V3-V4 regions of the 16S rRNA gene were used to construct the DNA library for sequencing. Paired-end sequencing of bacterial amplicons was performed on the Illumina MiSeq instrument (300 bp paired-end reads). Raw sequencing data have been deposited in the NCBI SRA database under BioProject accession number PRJNA739098 .

### Soil community co-existence analysis

Reads were processed using the UPARSE pipeline (http://drive5.com/usearch/manual/uparse_pipeline.html). The raw sequences were first trimmed to a length of 250 bp using the “fastx_truncate” command to discard shorter sequences. The paired-end reads were merged using the “fastq_mergepairs” command (less than three mismatches). High-quality sequences were then selected using the “fastq_filter” command (maximum error rate <0.5%) and dereplicated using the “derep_fulllength” command. The singletons were removed using the “unoise3” algorithm, and chimeric sequences were removed using the “uchime_ref” command with the RDP database (RDP training set v16) (https://www.drive5.com/usearch/manual/sintax_downloads.html). The remaining sequences were clustered into operational taxonomic units (OTUs) based on 97% sequence similarity. Taxonomy assignment of the OTUs was classified using the “sintax” algorithm (confidence threshold 0.6) with the RDP database. Finally, a rarefied OTU table was created using the USEARCH “otutab_norm” command at a depth of 10,000 reads per sample. The bacterial composition in the biofilm was visualized in Microsoft Office Excel 2019 to predict co-existence.

### Cultivation of the initial 11-species community

The 11-species biofilm was cultivated by mixing 4 mL of start inoculum with 400 mL of TSB and incubating at 30°C. Biofilms formed at the air-liquid interface were collected on days 0, 2, 4, 6, and 8. Each time point had eight biological replicates. The genomic DNA of the biofilm samples was extracted as previously described. 16S rDNA gene amplicon sequencing was conducted as described above.

### Initial 11-species SynCom composition analysis

Amplicon sequencing data were analyzed as described above. The difference in the analysis is that the generated sequences were not clustered and were directly used to create the amplicon sequence variant (ASV) table. The taxonomy of the ASVs was assigned to the species with a reference database consisting of the full 16S rDNA gene sequences of the 11 species. The ASVs were rarified using the USEARCH “otutab_rare” command at a depth of 10,000 reads per sample. Microbial co-occurrence networks were constructed to show the interactions among species during biofilm development. Data from all five time points were used to build the network. Spearman correlations among all taxa were calculated using the R psych package. Only edges with correlation scores >0.6 were kept (*P* < 0.05, false discovery rate [FDR] adjusted). Correlation networks were visualized via Gephi using the Fruchterman Reingold layout (55).

### Reduced SynComs biomass quantification

The five- and six-species biofilms were grown in 6-well microtiter plates (VWR Standard Multiwell Cell Culture Plates, Cat # 10861-554) inserted with 100 µm sterile nylon mesh cell strainers (Biologix Cat # 15-1100). Then, 10 mL of TSB liquid medium and 100 µL of start inoculum were added. The plates were incubated at 30°C to allow the biofilm to grow on top of the nylon mesh cell strainer. Biofilm biomass was defined by the wet weight and dry weight at 24, 36, and 48 h. The cell strainer was taken out of the well, and visible drops were removed with paper. The wet weight of the cell strainer was recorded. The cell strainer was then dried at 60°C for 12 h and weighed. Pellicle weight was the total weight minus the weight of the nylon mesh. Each treatment had six biological replicates.

### Reduced SynComs cell number quantification by qPCR

Strain-specific primers were designed for the selected six isolates. Genome comparison was performed using Roary (56) to find out the strain-specific single-copy genes of each isolate. Primers were designed to target these genes. qPCR was used to evaluate the specificity of the primers. Only primers that fulfill the following criteria were selected (Data S1): (i) selectively amplify target isolates but do not amplify non-cognate isolates. (ii) The CT values are low for targeting isolates and high for non-targeting isolates. The CT values for non-targeting isolates are similar to those of water. (iii) The melting curves display one peak for targeting isolates. The amplified fragments were ligated to PMD19T plasmids. Standard curves were generated using the plasmids containing corresponding fragments as templates (Table S1).

To quantify the cell numbers of each isolate within the reduced biofilms, 100 µL of the starting inoculum was grown in 6-well microtiter plates (VWR) with 10 mL of TSB medium. A 100-µm sterile nylon mesh cell strainer and a Spectra Mesh Woven Filter (Fisher Scientific, Spectrum 146488) were put inside. The mesh was manually cut into 1.5 cm^2^ squares and autoclaved. The mesh ensured an equal sampling of biofilm. After 24 or 36 h of pellicle development, the nylon mesh cell strainer was taken out, and the inner filter was transferred to a 1.5-mL microcentrifuge tube and stored at −80°C for the following DNA extraction. The genomic DNA of the biofilm samples was extracted using an E.Z.N.A. Bacterial DNA Kit (Omega Bio-tek, Inc.) following the manufacturer’s instructions. qPCR was performed with an Applied Biosystems Real-Time PCR Instrument. Reaction components are as follows: 7.2 µL H_2_O, 10 µL 2× ChamQ SYBR qPCR Master Mix (Vazyme), 0.4 µL 10 µM of each primer, and 2 µL template DNA. The PCR programs were carried out under the following conditions: 95°C for 10 min, 40 cycles of 95°C for 30 s, and 60°C for 45 s, followed by a standard melting curve segment. Each treatment had six biological replicates.

### Colony phenotype assay

The overnight culture was adjusted to an OD_600_ of 1. Then, 2 µL of cell suspension was spotted on the TSB or M9 glucose (0.2% glucose) agar plates and cultivated at 30°C. Photos were taken after 48 h of cultivation.

### Pair-wise assay

The direct competition of these isolates against each other was evaluated using the spot-on-lawn assay and the pair-wise spot assay. Spot-on-lawn assay: 5 mL of lawn species (OD_600_, ~0.02) grown in TSB medium was spread onto a 25-mL TSB plate (1.5% agar), and the extra cultures were removed by pipetting. Plates were dried for 20 min. Then, 5 µL of spot species (OD_600_, ~0.4) grown in TSB medium was spotted on the center of the lawn plates. Pair-wise spot assay: 5 µL of the dual-species (OD_600_, ~1) grown in TSB medium was spotted on the TSB plate (1.5% agar) at 5 mm between the center of each colony. Plates were grown at 30°C and imaged at 48 h. The experiments were performed twice; each experiment had three replicate plates.

### Growth curve assay

Growth in the TSB medium and Chr supernatant was evaluated by a growth curve assay. Chr supernatant was collected by growing 500 µL of Chr (OD_600_, ~1) in 50 mL TSB medium at 30°C and 170 rpm for 24 or 36 h and filter sterilizing it. Then, 2 µL of isolates (OD_600_, ~1) was inoculated into 200 µL of TSB medium or Chr supernatant in a 10 × 10 well honeycomb microplate. OD_600_ was measured every 30 min at 30°C with the Bioscreen C Automated Microbiology Growth Curve Analysis System. The growth was measured for 24 or 36 h. Each treatment has five replicates.

### Genome-scale metabolic modeling

The complete genome of the six isolates (Aci, Chr, Com, Ent, Pan, and Pse) was annotated using the RAST server (57). Genome-scale metabolic models of the six isolates were reconstructed using the CarveMe pipeline (58). The protein file was provided as an input file for CarveMe to generate a universal model using the following command: “carve genome.faa --output model.xml.” As four isolates (Aci, Ent, Pan, and Pse) were tested to grow in M9 glucose medium, this medium was used to perform gap-filling using the following command: “gapfill model.xml -m M9 -o new_model.xml.” The final model was exported as an SBML file. The quality of the metabolic models was assessed using MEMOTE (59) and provided as an HTML file. The metabolic interaction potential and metabolic resource overlap for each community were analyzed using SMETANA (37, 38). The global metabolic interaction analysis of the SynComs was performed using the following command: “smetana *.xml #.xml --mediadb M9.tsv -m M9 -g.” The detailed metabolite exchange of the full community was analyzed using the following command: “smetana *.xml #.xml --mediadb M9.tsv -m M9 -d.” The simulated cross-feeding results were summarized as the SMETANA score, which estimates the strength of metabolic exchange (37).

### Experimental validation of metabolic models

The potential growth promotion of the bacterial metabolites to another species was evaluated using a spent medium growth curve assay. Donor bacteria were grown in the M9 medium with 0.2% glucose until the glucose was under the limit of detection. The consumption of glucose was measured using the Glucose GO Assay Kit (Sigma). The cell culture was spun down, and then the spent medium was filter-sterilized and directly used as the medium for the growth curve assay. Then, 2 µL of recipient species (OD_600_, ~1) was inoculated into 200 µL of spent medium or M9 glucose medium in a 10 × 10 well honeycomb microplate. OD_600_ was measured every 30 min at 30°C for 48 h. Each treatment has five replicates. The growth rate and growing capacity (maximum population size) are compared.

Metabolites that are predicted to be donated by Pan and received by other isolates were tested by growth assay. The compounds were provided as sole carbon sources in M9 minimal medium at a concentration of 0.2 g/L or at maximum solubility if the water solubility was lower than 0.2 g/L. Then, 3 µL of recipient species (OD_600_, ~1) was inoculated into 300 µL of medium in a 48-well microplate. The microplate was incubated at 30°C and 220 rpm for 48 h. Then, 200 µL of cell suspension was transferred into a 96-well microplate, and OD_600_ was measured. Twenty compounds were tested by this method (see Fig. S9). The mixture indicated that the 20 compounds were mixed at equal volume. Each treatment has three replicates. OD_600_ higher than 0.1 is defined as growth.

### Carbon source metabolic activity measurement

PM1 (BIOLOG Cat #12111) and PM2 (BIOLOG Cat #12112) phenotypic microarrays were used to assess the carbon source utilization ability of the community members (60). The assays were performed following the manufacturer’s instructions. Briefly, 100 µL of diluted cell suspension of each species mixed with the BiOLOG redox dyes (the final cell density is 85% transmittance in the Biolog Turbidimeter) was added to each well of the PM plates. Water mixed with dyes was used as a negative control. All plates were then incubated at 30°C for up to 48 h. If the species could utilize the carbon source in a well, the colorless tetrazolium dye would be reduced to purple formazan by cell respiration. The color changes were measured by an endpoint absorbance at 590 nm with a microplate reader. The variable level of color changes indicates the carbon source of the metabolic activity.

### Statistical analysis and figures

Data were analyzed using R v4.1.3 (61) in the RStudio v2022.02.0 + 443. Statistical analysis methods were described in figure legends. Welch’s ANOVA and Games-Howell post hoc tests were used to identify statistically significant differences between biofilm weight and cell numbers in different communities. Plots were generated using Microsoft Office Excel 2019 (stacked bar plot), R ggplot2 (62), ggpubr (63), ggalluvial (64), pheatmap (65) packages, and Adobe Illustrator CC 2020 (Adobe Inc.). Schematic diagrams were generated using BioRender (https://biorender.com/).

## Data Availability

All the data needed to evaluate the conclusions in this paper are provided in the supplemental material. Detailed information on strain-specific primers is provided in Data S1. The data used to generate the standard curves for qPCR are provided in Table S1. Metabolic models and MEMOTE scores are provided in Data S2. The data used in the main figures are provided in Table S2. The data used in the supplemental figures are provided in Table S3. Raw sequencing data have been deposited into the NCBI SRA database, as described above.

## SUPPLEMENTAL MATERIAL

Data S1 msystems.01045-23-s0001.pdf Primer specificity.

Supplemental figures msystems.01045-23-s0002.pdf Figures S1 to S10.

Supplemental information guidance msystems.01045-23-s0003.docx Captions to Data S1 and Tables S1 to S3; link to Data S2.

Table S1 msystems.01045-23-s0004.xlsx Standard curve.

Table S2 msystems.01045-23-s0005.xlsx Main figure data.

Table S3 msystems.01045-23-s0006.xlsx Data for supplemental figures.

ASM does not own the copyrights to Supplemental Material that may be linked to, or accessed through, an article. The authors have granted ASM a non-exclusive, world-wide license to publish the Supplemental Material files. Please contact the corresponding author directly for reuse.
