# Supplementary material for: Metabolic interactions affect the biomass of synthetic bacterial biofilm communities
Source: mSystems. 2023 Nov 16;8(6):e01045-23. doi: 10.1128/msystems.01045-23 (PMC10734490; doi:10.1128/msystems.01045-23)
Supplement: Data S1 — Primer specificity. [file msystems.01045-23-s0001.pdf]

Plasmid: PMD19T
Competent cell: E.coli DH5α

Primers: Aci_F, Aci _R

Amplification plot

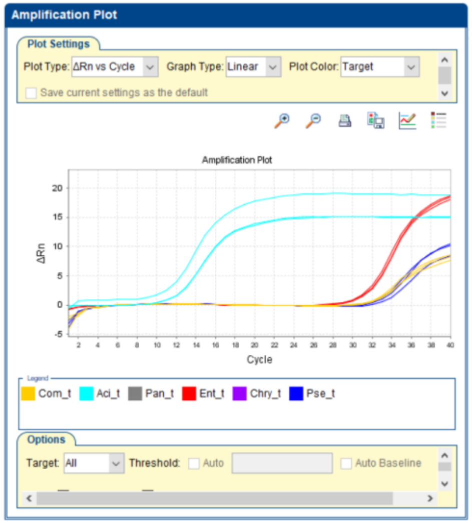

Legend: DNA template

Melt curve

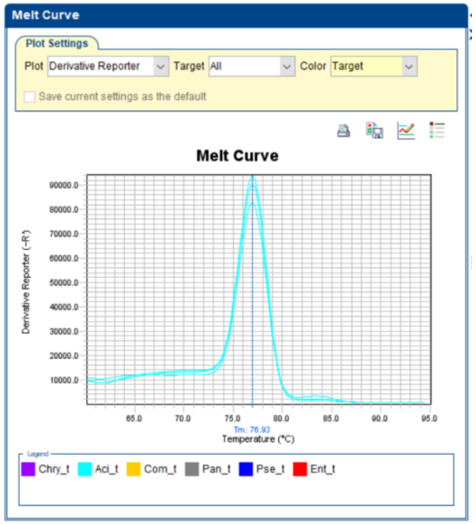

Template: genomic DNA of **Aci**

Aci_F: ATTTAGTATCTGGTGAAGTCATCCGTA
Aci_R: CCGACAAATAAAGCTTGAGTAACTCC
Product length: 92 bp
Target gene:
>lcl|NZ_CP046536.1_cds_WP_000428564.1_58 [locus_tag=GOD87_RS00290] [protein=DUF1003 domain-containing protein] [protein_id=WP_000428564.1] [location=61045..61746] [gbkey=CDS]
Standard curve:
lg[DNA copies]/ul=-0.2897*CT+11.9310

Standard curve

Template: plasmid containing Aci fragment

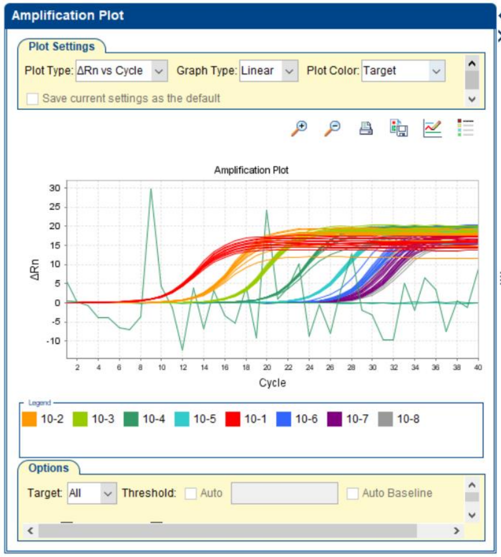

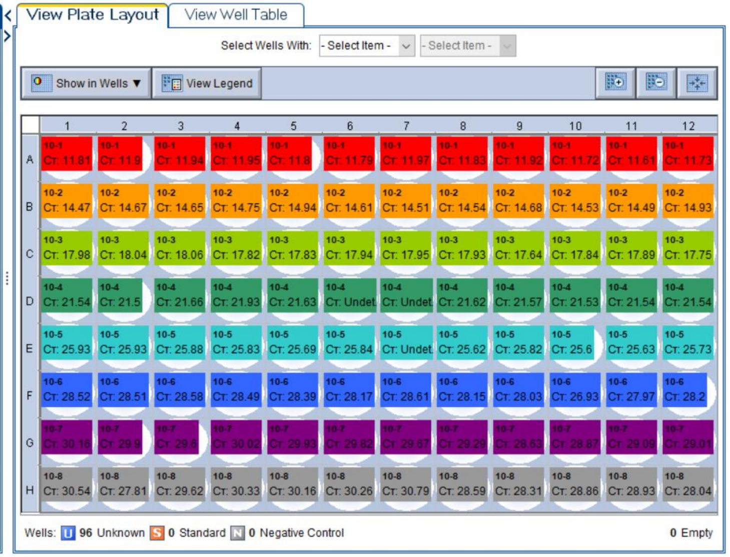

Concentration of DNA template decrease by tenfold

Primers: Com_F, Com_R

Amplification plot

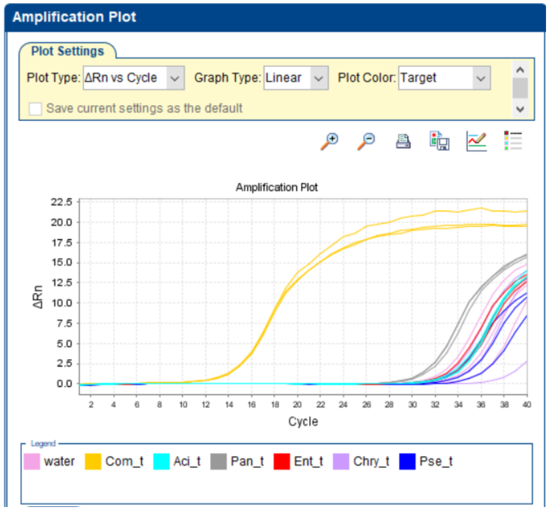

Legend: DNA template

Melt curve

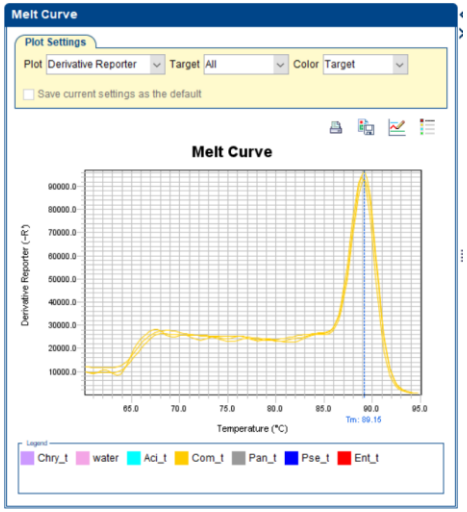

Template: genomic DNA of **Com**

Com_F: CTCAAAACCAGTGTGATCGTGGAA
Com_R: TATTGCCCATCAGCAGAGTGTAGC
Product length: 109 bp
Target gene:
>lcl|NZ_CP083451.1_cds_WP_224152861.1_192 [locus_tag=LAD35_RS00960] [protein=tripartite tricarboxylate transporter substrate binding protein] [protein_id=WP_224152861.1]
Standard curve:
lg[DNA copies]/ul=-0.2785*CT+12.5029

Standard curve

Template: plasmid containing **Com** fragment

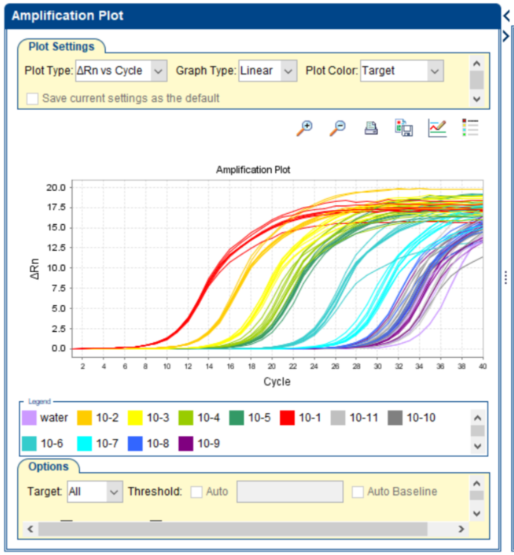

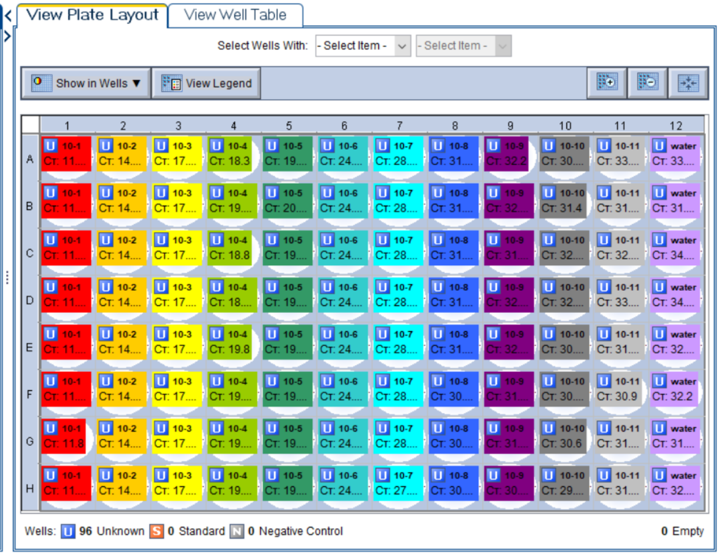

Concentration of DNA template decrease by tenfold

Primers: Chr_F, Chr_R

Amplification plot

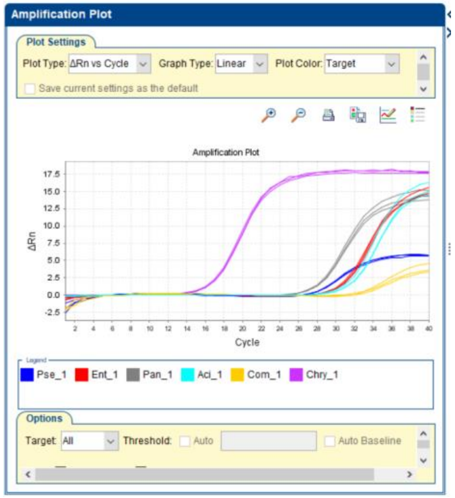

Legend: DNA template

Melt curve

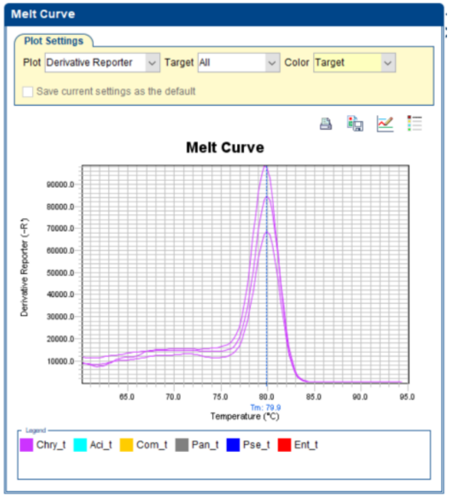

Template: genomic DNA of **Chr**

Primer information:

Chr_F: GAACATCAGTTATCTTGTGAGCGGTA

Chr_R: CATACAGGCTCCCATTCCTATTGTG

Product length: 94 bp

Target gene:

>lcl|NZ_CP084347.1_cds_WP_225717821.1_11 [locus_tag=KB553_RS00055] [protein=MFS transporter] [protein_id=WP_225717821.1] [location=9574..11166] [gbkey=CDS]

Standard curve:

lg[DNA copies]/ul=-0.2957*CT+12.4720

Standard curve

Template: plasmid containing **Chr** fragment

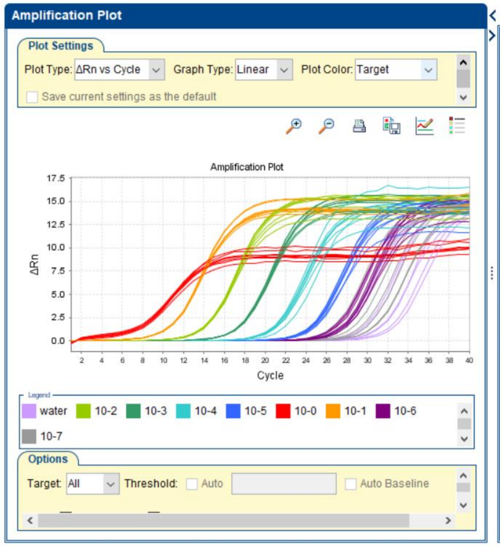

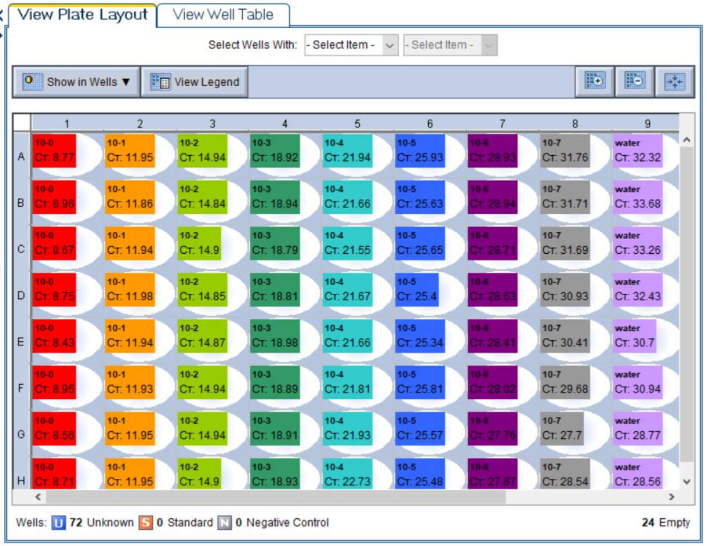

View Plate Layout | View Well Table

Select Wells With: - Select Item - | - Select Item -

Show in Wells ▼ | View Legend

	1	2	3	4	5	6	7	8	9
A	10-0 CT: 8.77	10-1 CT: 11.95	10-2 CT: 14.94	10-3 CT: 18.92	10-4 CT: 21.94	10-5 CT: 25.93	10-6 CT: 28.93	10-7 CT: 31.76	water CT: 32.32
B	10-0 CT: 8.96	10-1 CT: 11.86	10-2 CT: 14.84	10-3 CT: 18.94	10-4 CT: 21.66	10-5 CT: 25.63	10-6 CT: 28.94	10-7 CT: 31.71	water CT: 33.68
C	10-0 CT: 8.67	10-1 CT: 11.94	10-2 CT: 14.9	10-3 CT: 18.79	10-4 CT: 21.55	10-5 CT: 25.65	10-6 CT: 28.71	10-7 CT: 31.69	water CT: 33.26
D	10-0 CT: 8.75	10-1 CT: 11.98	10-2 CT: 14.85	10-3 CT: 18.81	10-4 CT: 21.67	10-5 CT: 25.4	10-6 CT: 28.63	10-7 CT: 30.93	water CT: 32.43
E	10-0 CT: 8.43	10-1 CT: 11.94	10-2 CT: 14.87	10-3 CT: 18.98	10-4 CT: 21.66	10-5 CT: 25.34	10-6 CT: 28.41	10-7 CT: 30.41	water CT: 30.7
F	10-0 CT: 8.95	10-1 CT: 11.93	10-2 CT: 14.94	10-3 CT: 18.89	10-4 CT: 21.81	10-5 CT: 25.81	10-6 CT: 28.02	10-7 CT: 29.68	water CT: 30.94
G	10-0 CT: 8.56	10-1 CT: 11.95	10-2 CT: 14.94	10-3 CT: 18.91	10-4 CT: 21.93	10-5 CT: 25.57	10-6 CT: 27.76	10-7 CT: 27.7	water CT: 28.77
H	10-0 CT: 8.71	10-1 CT: 11.95	10-2 CT: 14.9	10-3 CT: 18.93	10-4 CT: 22.73	10-5 CT: 25.48	10-6 CT: 27.87	10-7 CT: 28.54	water CT: 28.56

Wells: U 72 Unknown S 0 Standard N 0 Negative Control 24 Empty

Concentration of DNA template decrease by tenfold

Primers: Ent_F, Ent_R

Amplification plot

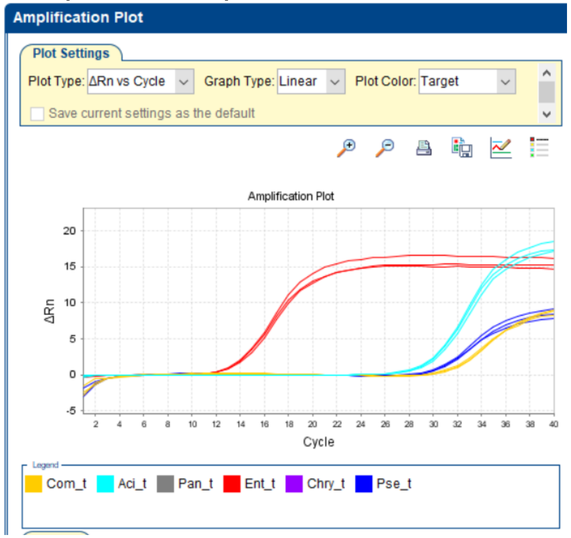

Legend: DNA template

Melt curve

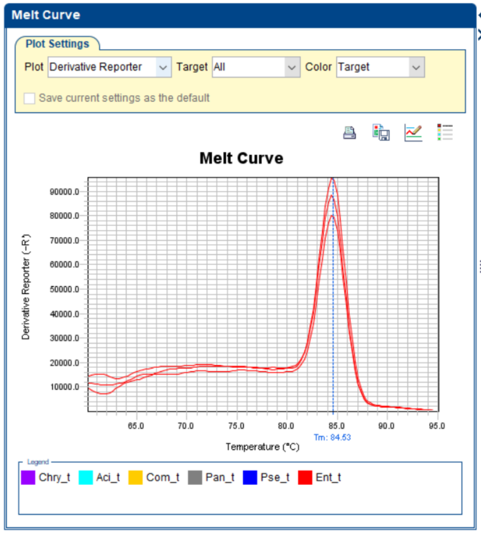

Template: genomic DNA of **Ent**

Ent_F: AGCGTTACAGCAGCTACAGGATATTCACC

Ent_R: CTTTTCACCATCACCCCATCCCTCGGTA

Product length: 95 bp

Target gene:

>lcl|NZ_CP083403.1_cds_80 [locus_tag=K9O83_RS00400] [protein=class I SAM-dependent methyltransferase] [pseudo=true] [partial=5'] [location=<76927..77040] [gbkey=CDS]

Standard curve:

lg[DNA copies]/ul=-0.3036*CT+12.5616

Standard curve

Template: plasmid containing **Ent** fragment

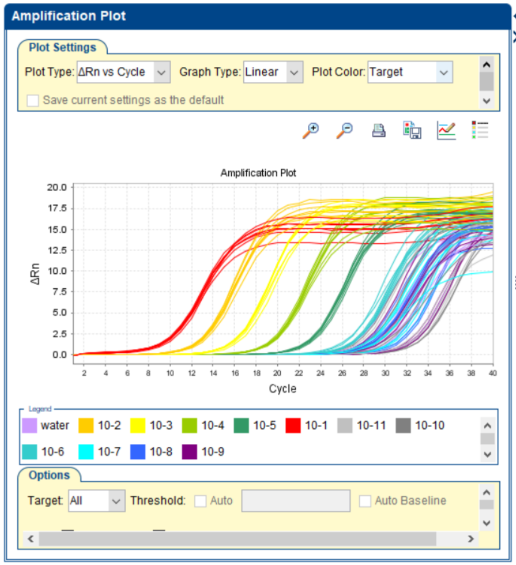

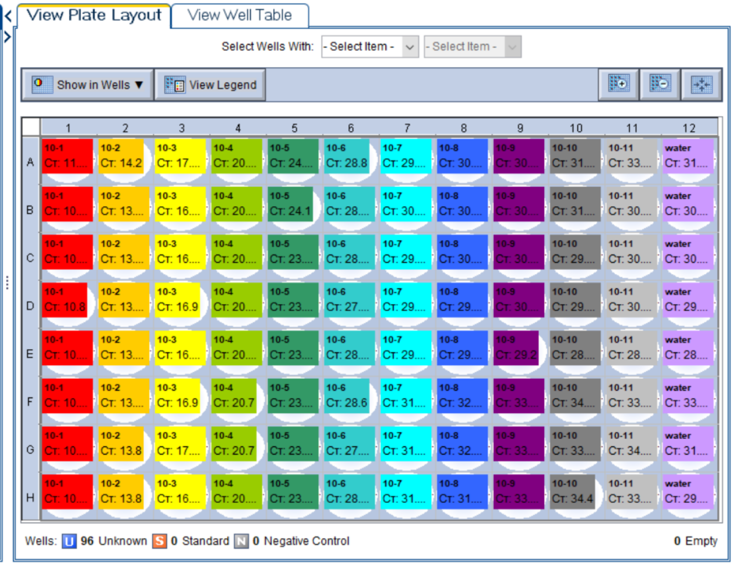

Concentration of DNA template decrease by tenfold

Primers: Pan_F, Pan_R

Amplification plot

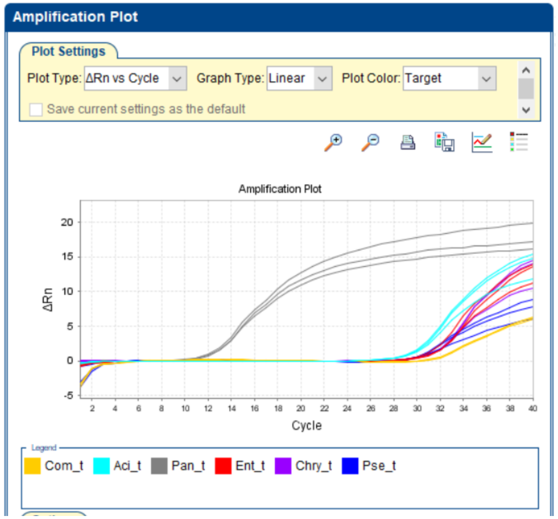

Legend: DNA template

Melt curve

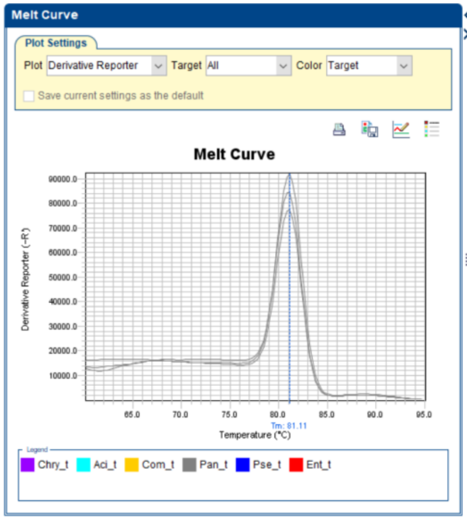

Template: genomic DNA of **Pan**

Pan_F: TTAACATCGAAAAGCCTTCCCACCGTA
Pan_R: ATTCATCAGAAGCGCATGTATTACACT
Product length: 101 bp
Target gene:
>lcl|NZ_CP083448.1_cds_WP_039382012.1_226 [locus_tag=LAC65_RS01220] [protein=hypothetical protein] [protein_id=WP_039382012.1] [location=272583..273698] [gbkey=CDS]
Standard curve:
lg[DNA copies]/ul=-0.3140*CT+12.9448

Standard curve

Template: plasmid containing **Pan** fragment

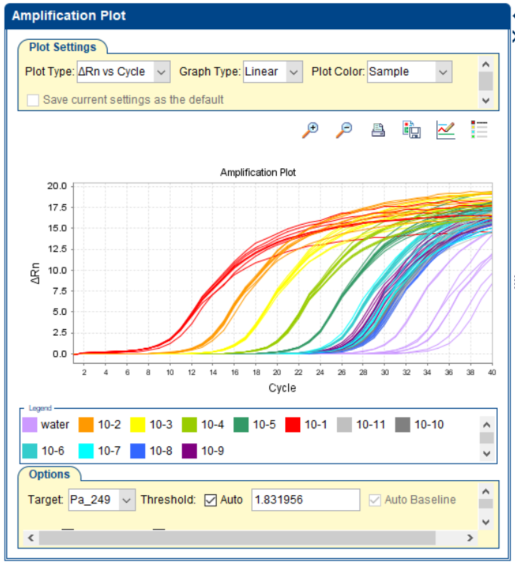

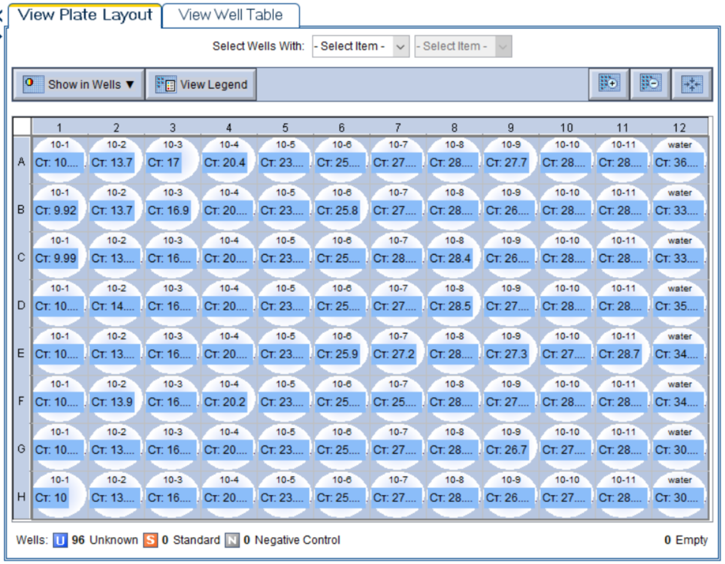

→ Concentration of DNA template decrease by tenfold

Primers: Pse_F, Pse_R

Amplification plot

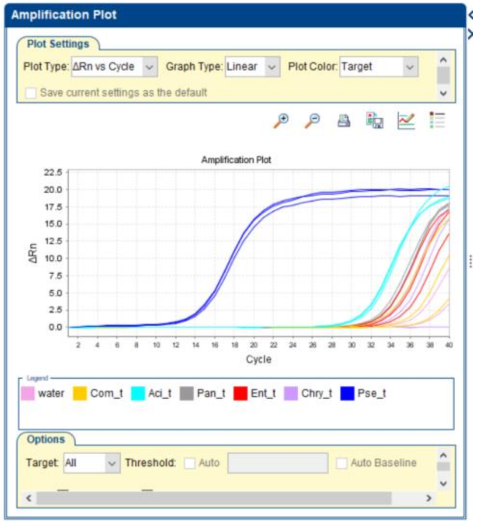

Legend: DNA template

Melt curve

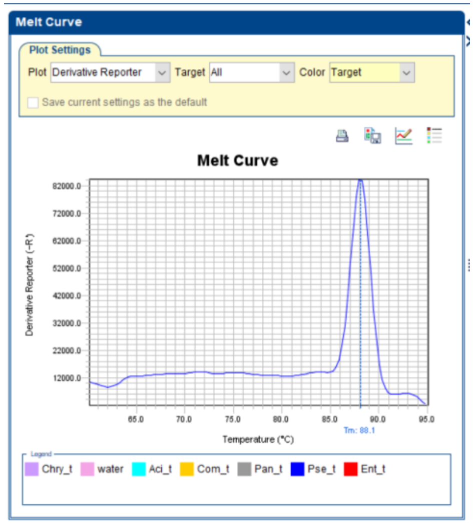

Template: genomic DNA of **Pse**

Pse_F: GAAATTCATCTTCGAACACAGCACAC
Pse_R: CTAGCTAACGGGGTTAAGTGCTTC
Product length: 124 bp
Target gene:
>lcl|NZ_CP046538.1_cds_WP_156714731.1_565 [locus_tag=GOM96_RS02845] [protein=efflux RND transporter permease subunit] [protein_id=WP_156714731.1] [location=646415..648859] [gbkey=CDS]
Standard curve:
lg[DNA copies]/ul=-0.2807*CT+12.2515

Standard curve

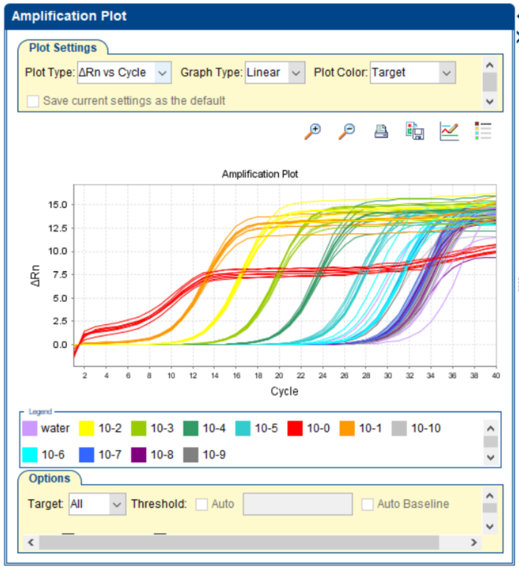

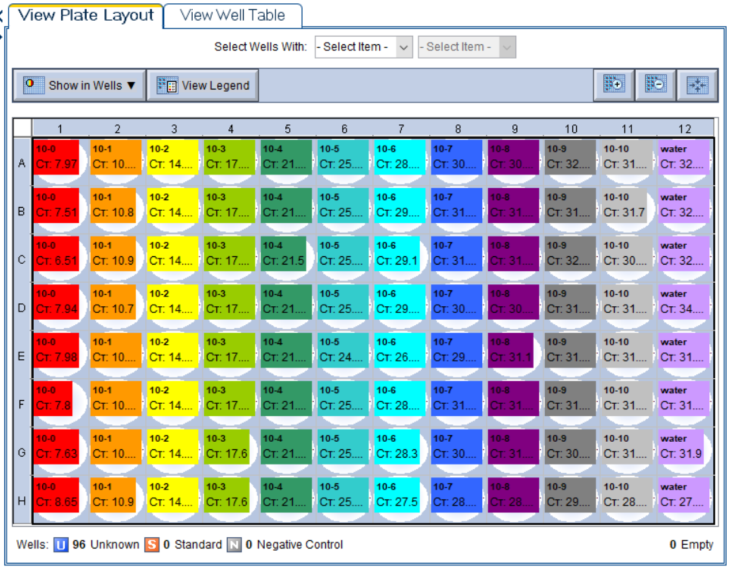

Concentration of DNA template decrease by tenfold
