# Supplementary material for: Metabolic interactions affect the biomass of synthetic bacterial biofilm communities
Source: mSystems. 2023 Nov 16;8(6):e01045-23. doi: 10.1128/msystems.01045-23 (PMC10734490; doi:10.1128/msystems.01045-23)
Supplement: Supplemental figures — Figures S1 to S10. [file msystems.01045-23-s0002.pdf]

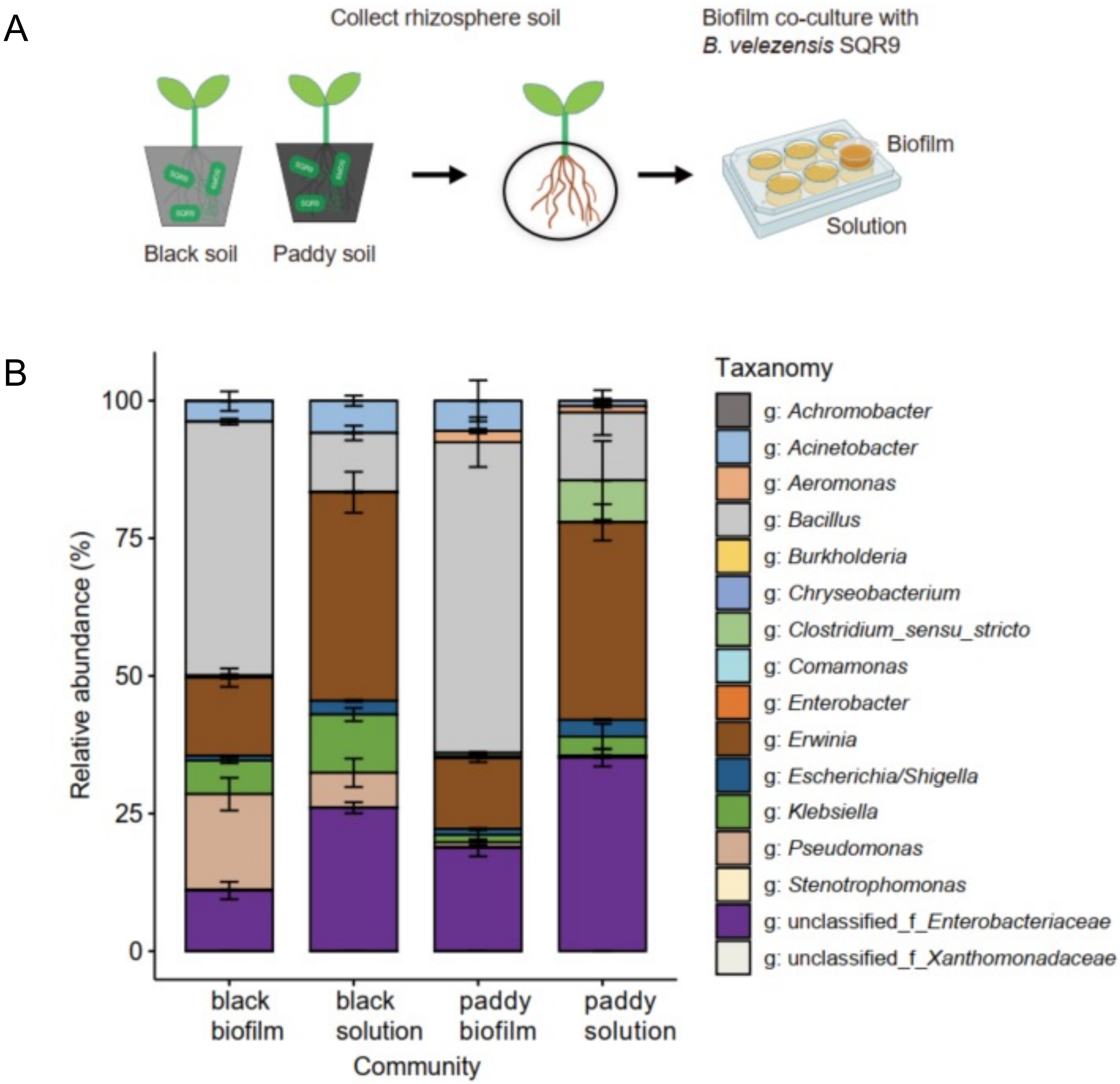

Figure S1. Origin of the initial 11 isolates. (**A**) Schematic diagram of the experimental setup. Rhizospheric black soil and paddy soil were collected from cucumber plants. The soil microbiota were co-cultivated with *B. velezensis* SQR9 at 30°C in TSB medium to form pellicle biofilms. After 24 hours of cultivation, the pellicle and the solution underneath were collected separately. The samples were sent for 16S rDNA amplicon sequencing. (**B**) Microbiome composition of the biofilm and solution. Data presented are the mean ±sd. n =3. Based on OTU clustering and the RDP database taxonomic classification, 15 genera and 2 families were identified. Eleven matching isolates were selected from our laboratory bacteria collection (Sun et al., 2021).

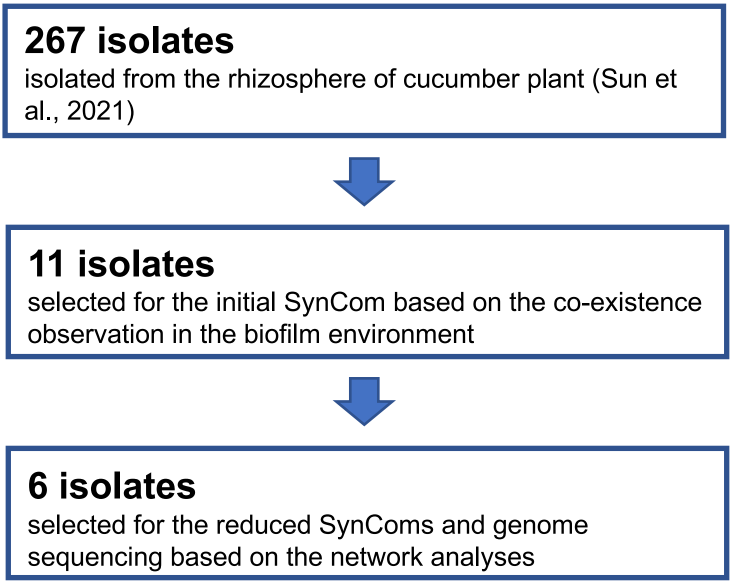

Figure S2. A flow chart of isolates used for the study, their origin and selection criteria.

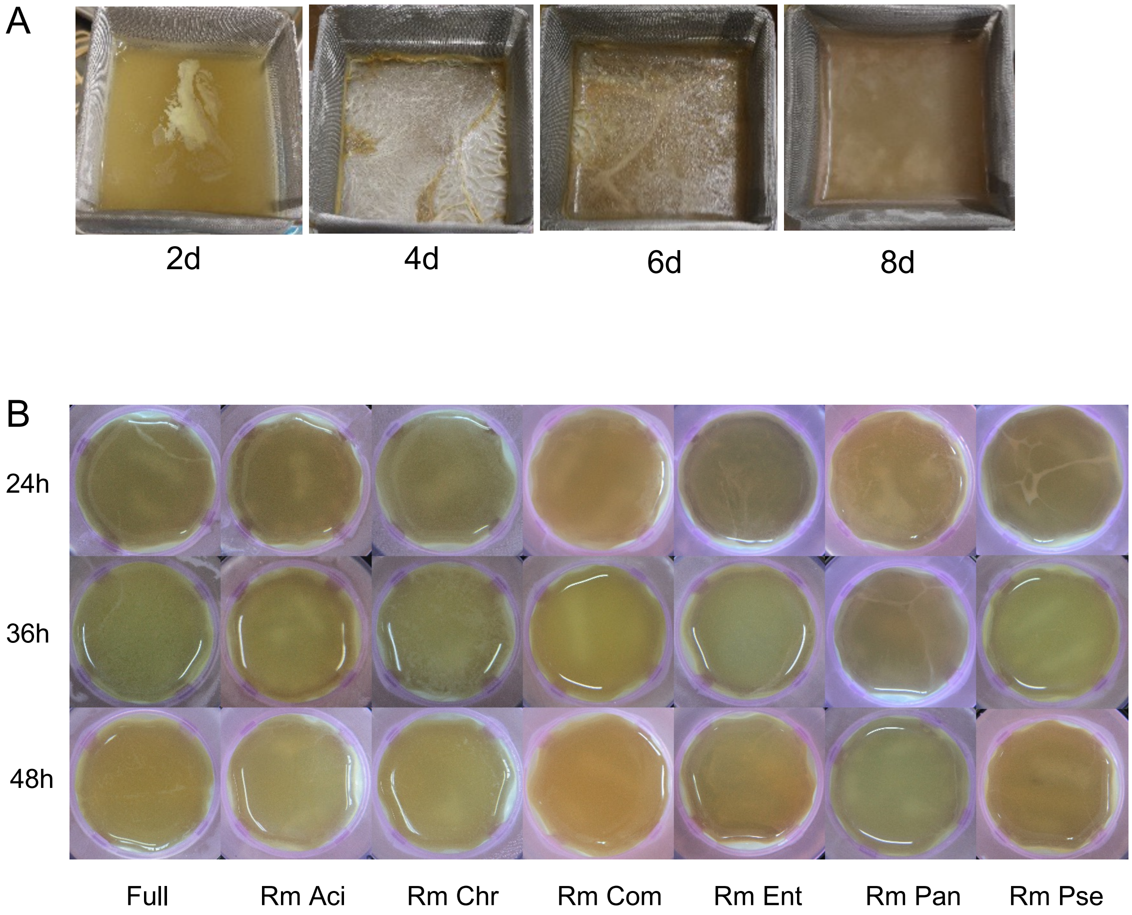

Figure S3. Biofilm development of the 11-isolates' community (**A**) and reduced communities (**B**). The 11-isolates' community was cultivated in 400 ml system, the reduced communities were cultivated in 10 ml system. Three major events in biofilm formation: aggregation (bacteria aggregate to each other), growth (bacteria aggregates expand by growth and recruitment of surrounding cells), and disaggregation (bacteria leave the biofilm as aggregates and single cells) (Sauer *et al*. 2022).

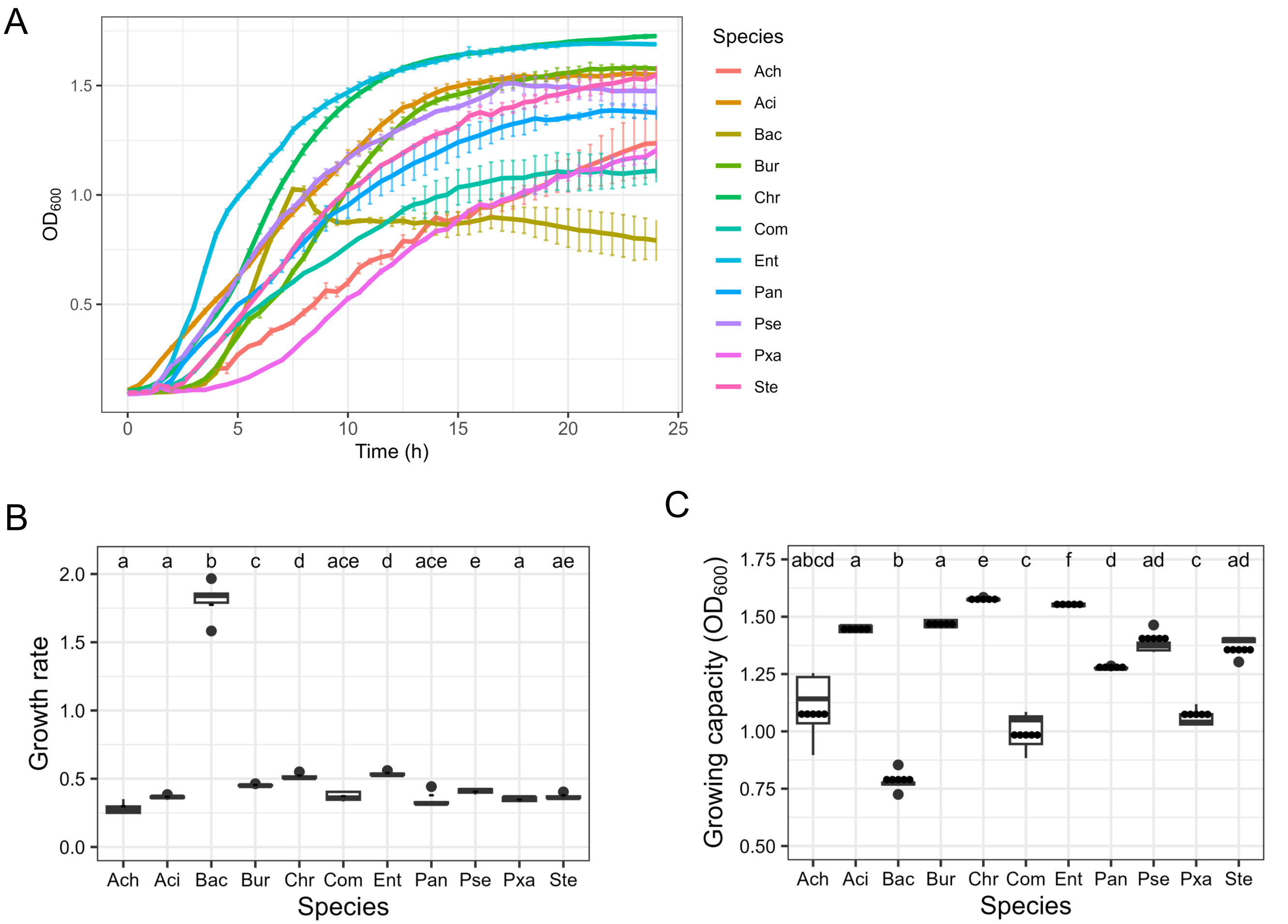

Figure S4. **Growth in TSB medium**. (A) Growth curves. Data presented are the mean ±sd. n =5. (**B**) The intrinsic growth rate of the population, r, is the growth rate that would occur if there were no restrictions imposed on total population size. (**C**) Growing capacity, k, indicates the maximum population size. R and k were calculated using the *Growth Curver* R package. Different letters indicate significant differences by Welch's ANOVA and Games Howell post hoc test, $p < 0.05$, n = 5.

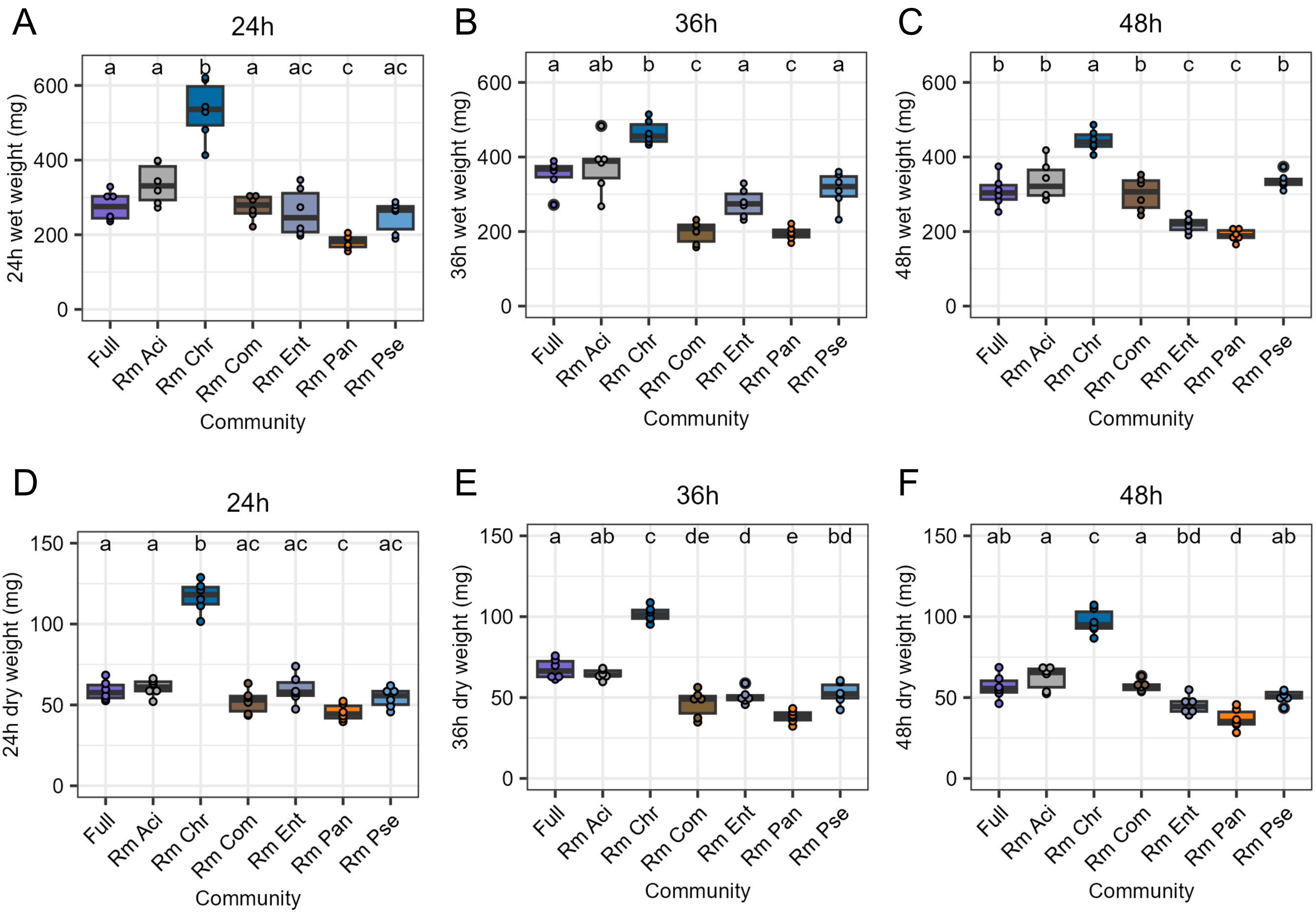

Figure S5. Biofilm weight of the reduced SynComs. Wet weight at 24h (**A**), 36h (**B**), 48h (**C**). Dry weight at 24h (**D**), 36h (**E**), 48h (**F**). Boxes depict the first and third quartile, the horizontal line the median, and whiskers the 1.5 interquartile range. Data points represent replicates. The data are normally distributed, shaprio test. The groups have unequal variance, Levene test. Different letters indicate significant differences by Welch's ANOVA and Games Howell post-hoc test, $p < 0.05$, n = 6.

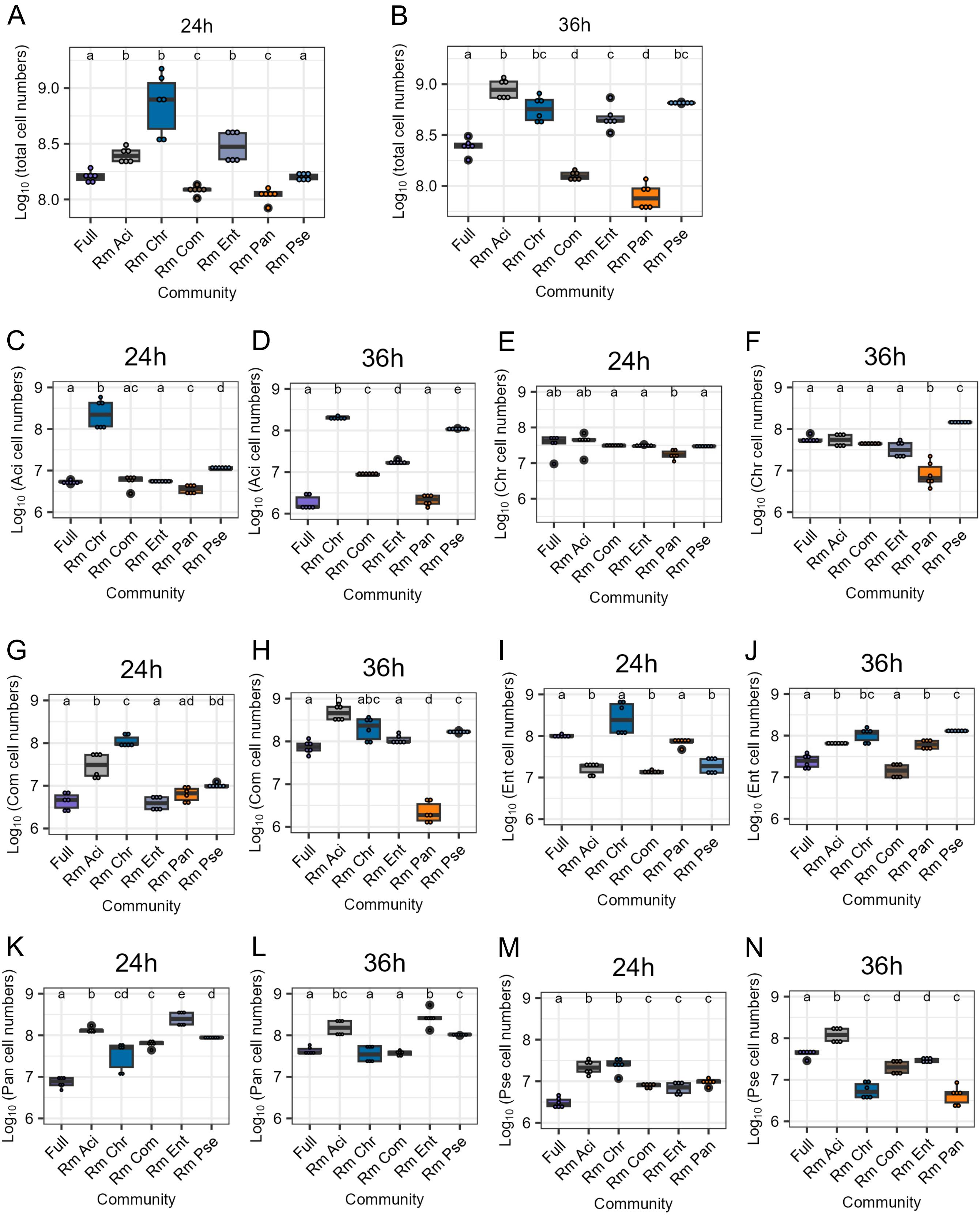

Figure S6. Cell numbers in different SynComs. (**A-B**) Total cell numbers. (**L-N**) Cell numbers of Aci, Chr, Com, Ent, Pan, Pse in different communities. Boxes depict the first and third quartile, the horizontal line the median, and whiskers the 1.5 interquartile range. Data points represent replicates. The data are normally distributed, shaprio test. The groups have unequal variance, Levene test. Different letters indicate significant differences by Welch's ANOVA and Games Howell post hoc test, $p < 0.05$, n = 6.

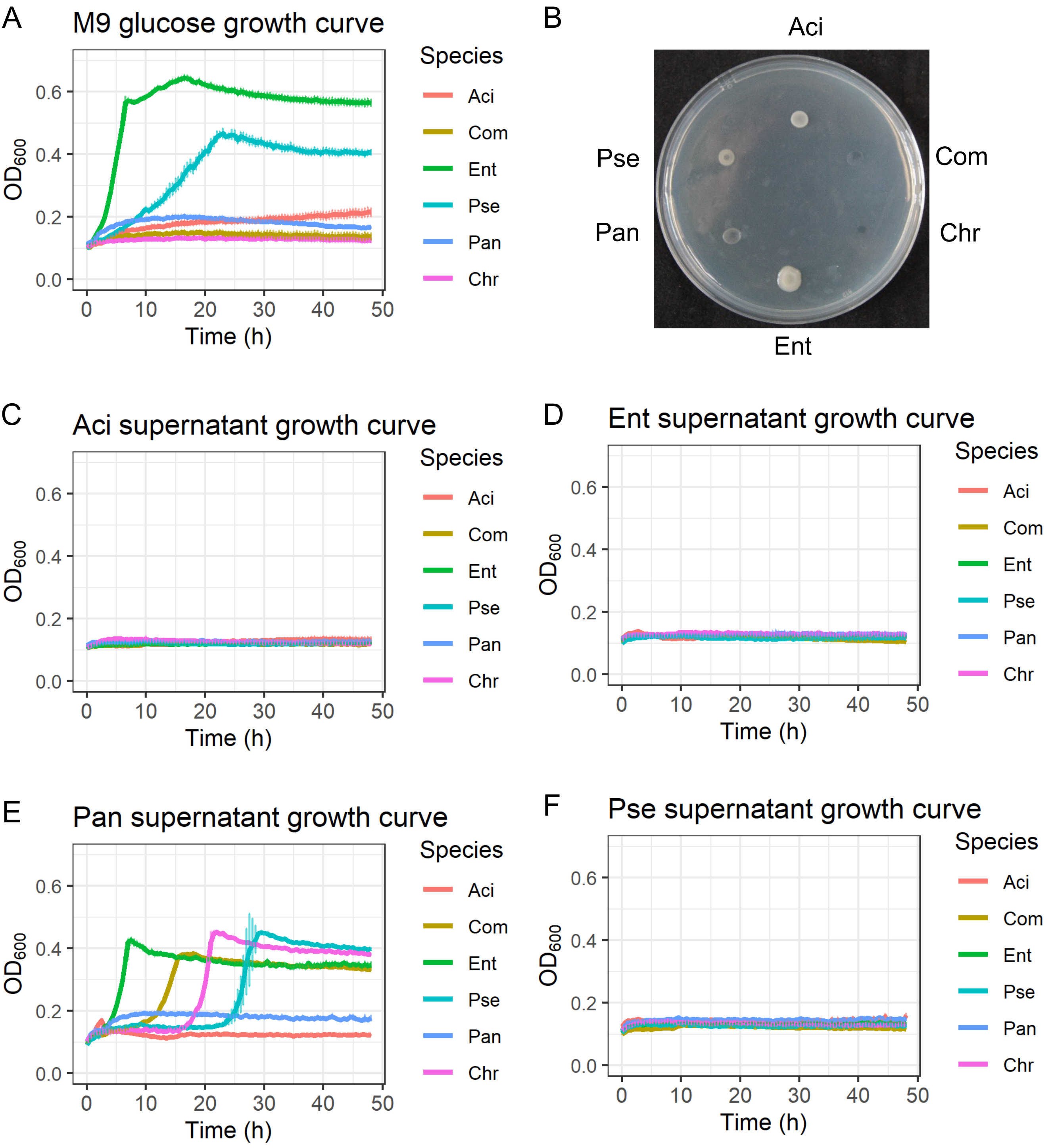

Figure S7. Growth curves. (**A**) Growth curves in M9 medium with 0.2% glucose as carbon source. (**B**) Colony phenotype on M9 glucose medium. (**C**) Growth curves in Aci spent medium. (**D**) Growth curves in Ent spent medium. (**E**) Growth curves in Pan spent medium. (**F**) Growth curves in Pse spent medium. Data presented are the mean ±sd. n =5.

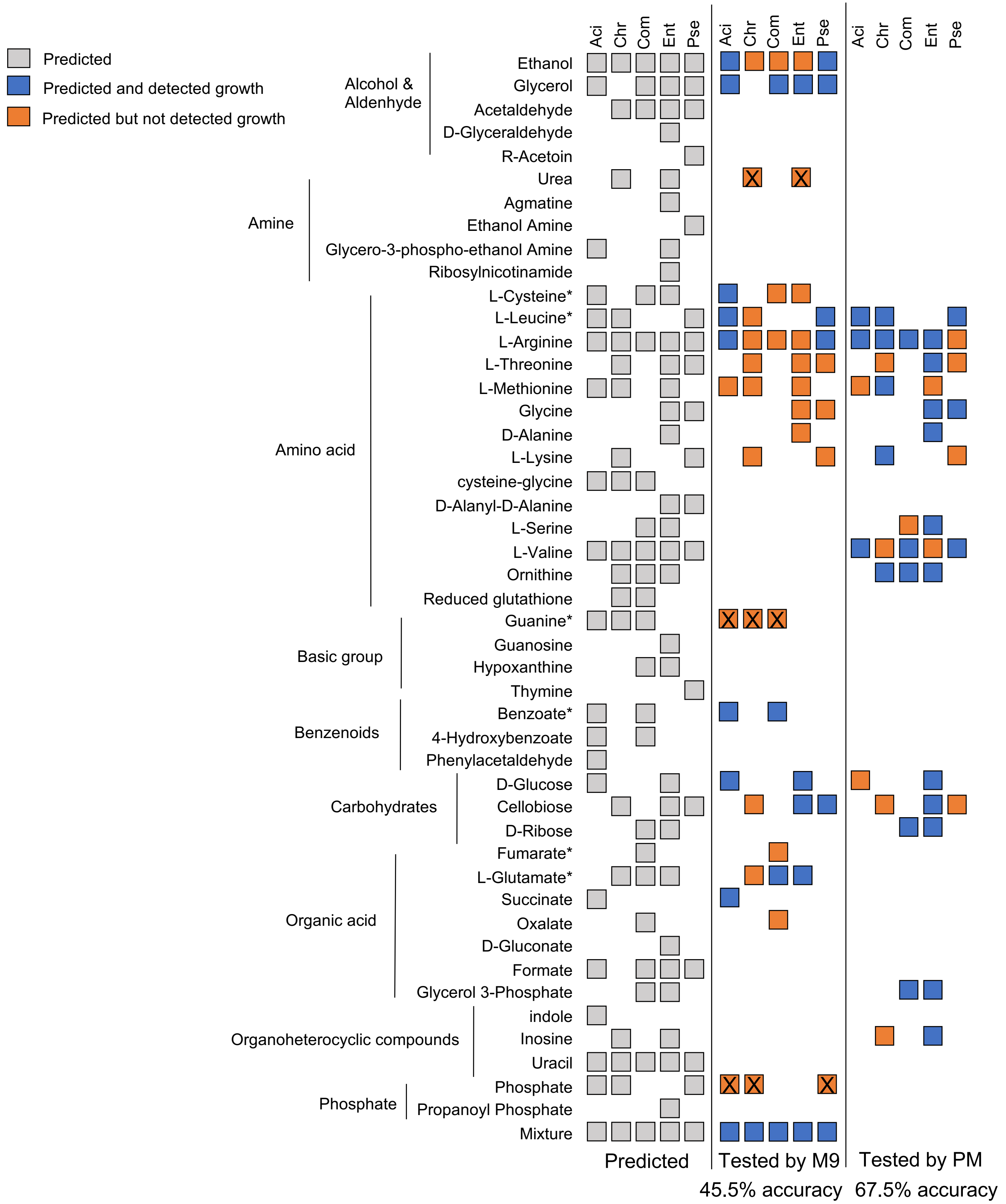

Figure S8. **Validation of metabolic modelling**. "Predicted" indicates the compound was predicted to be secreted by Pan and utilized by other isolates by metabolic models. "Tested by M9" indicates that the compound was provided as sole carbon source of M9 medium to test the growth of isolates. The compounds were provided at a concentration of 0.2g/L. "*" indicates that solubility of the compound in water is lower than 0.2g/L, thus it was tested at maximum solubility. "X" indicates that the compound is not a carbon source. OD_{600} higher than 0.1 was defined as growth. N=3. "Tested by PM" indicates that growth of isolates in the compound was tested by phenotype microarray assay. Omnilog value higher than 20 was defined as growth. n = 1. Accuracy is calculated as the number of "Predicted and detected growth" divided by the number of carbon sources tested. Note that calculation does not take into account mixture and compounds that are not carbon sources.

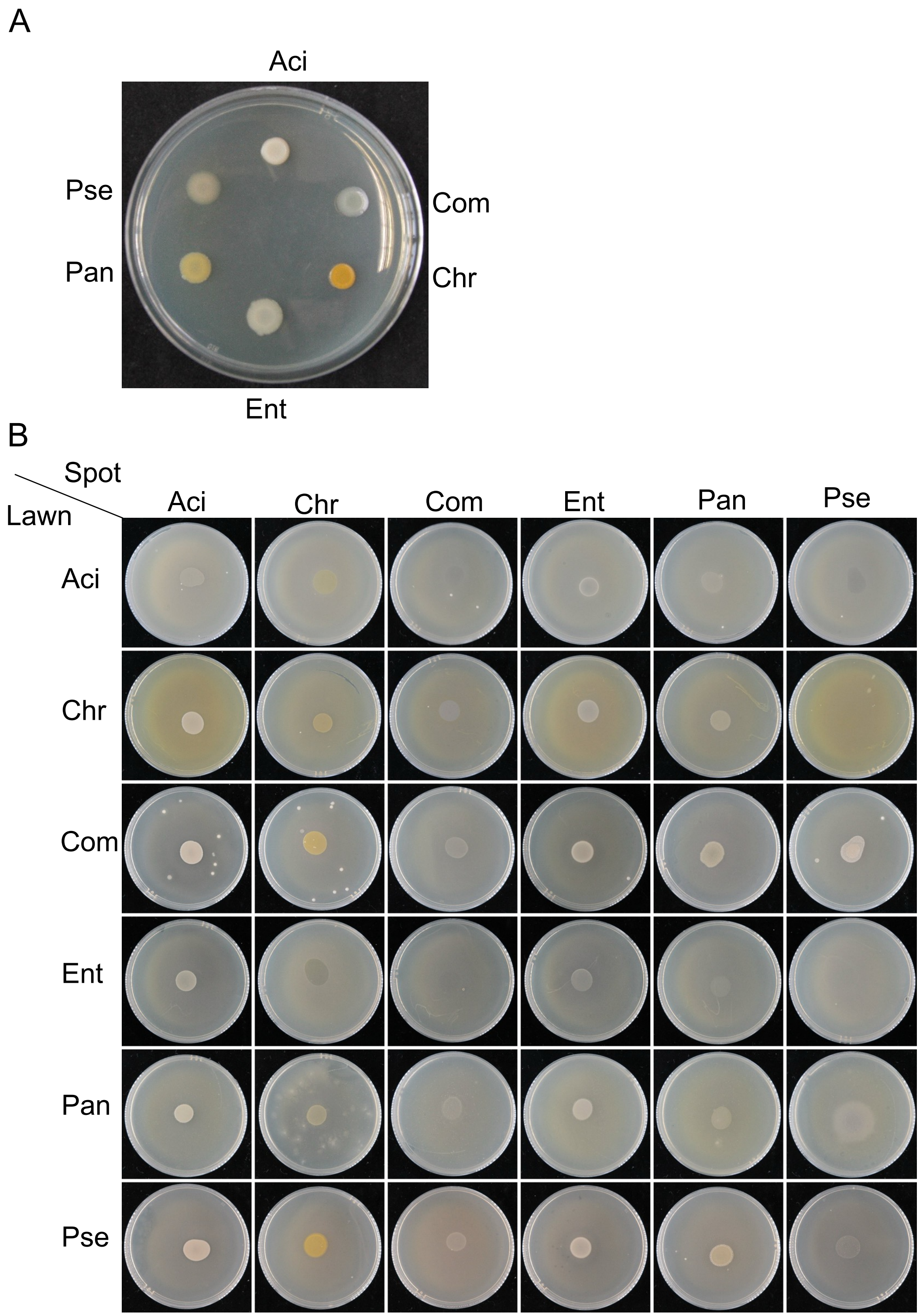

Figure S9. Colony phenotype. (**A**) Colony phenotype on TSB medium. (**B**) Spot-on-lawn phenotype. The lawn species were spread on TSB agar plates at an OD_{600} of 0.02 and dried, 5 µl of the spot species were spotted on the center at an OD_{600} of 0.4. The plate diameter is 6 cm. Photos were taken after 48 h incubation at 30°C.

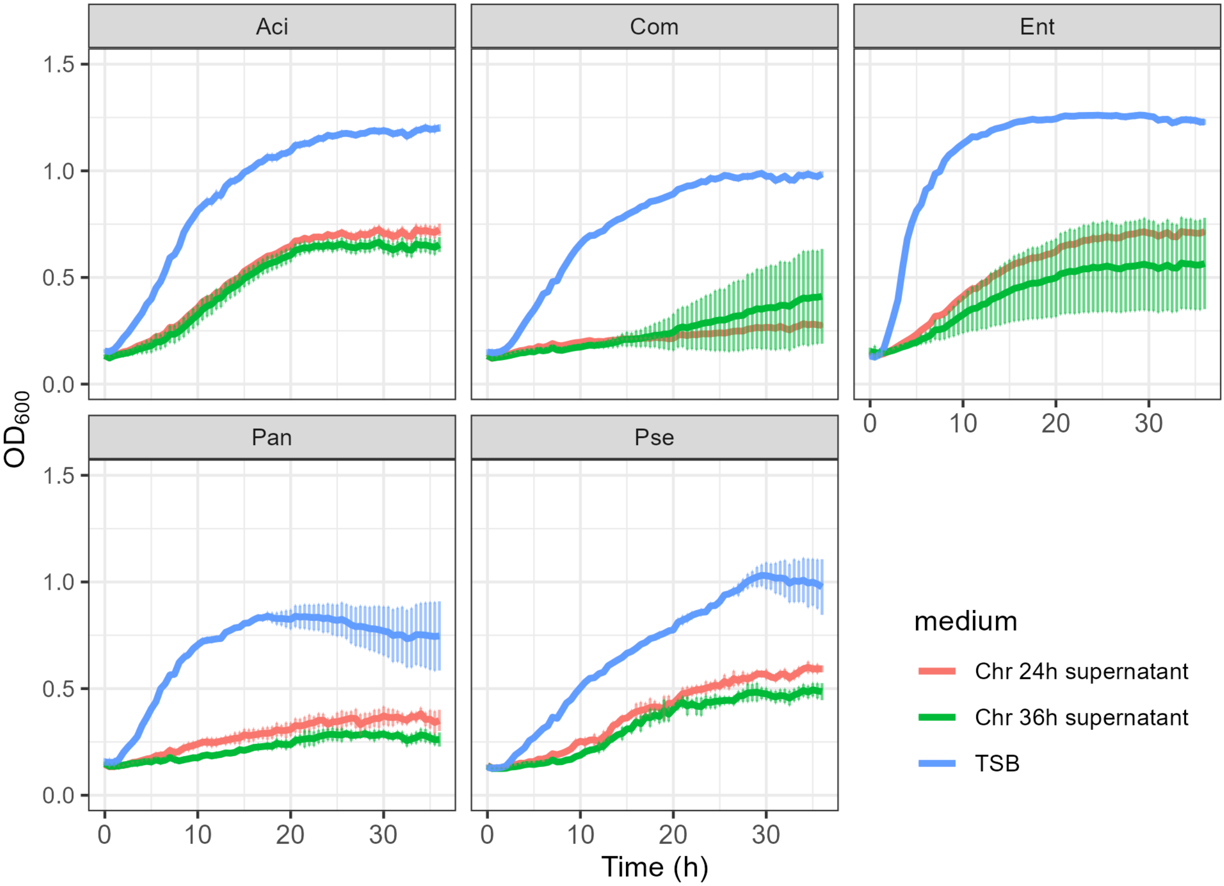

Figure S10. Growth curves in Chr supernatant. The supernatant was obtained by growing Chr in TSB medium and filter sterilizing it.
