# Supplementary material for: Metabolic interactions affect the biomass of synthetic bacterial biofilm communities
Source: mSystems. 2023 Nov 16;8(6):e01045-23. doi: 10.1128/msystems.01045-23 (PMC10734490; doi:10.1128/msystems.01045-23)
Supplement: Supplemental information guidance — Captions to Data S1 and Tables S1 to S3; link to Data S2. [file msystems.01045-23-s0003.docx]

**Supplementary Data**

**Supplementary Data 1.** PDF file containing detailed information on strain-specific primers. This includes plasmid used to carry the target sequences, primer sequences, product length, targeting genes, target gene function, target location on the genome, standard curves, and validation of the specificity of primers.

**Supplementary Data 2.** Metabolic models and MEMOTE score. **“Protein file folder”** includes amino acid sequence (.faa file) used for reconstruction of metabolic models. **“Draft model folder”** includes draft metabolic models (.xml file) and evaluation results by MEMOTE (.html file). “M9 gap filling model folder” includes metabolic models that were undergone gap filling procedure using the M9 medium and evaluation results by MEMOTE

Download in Github:

https://github.com/JiyuXie/DATA2/blob/main/Supplementary%20Data%202%20Metabolic%20models%20and%20Memote%20score.zip

**Supplementary Table 1.** Tables showing the process of calculating standard curves of strain-specific primers.

**Supplementary Table 2.** Tables containing the data used in the main figures. **Sheet 1,** Summary table of bacterial relative abundance in the 11-species biofilm community from 0d to 8d. **Sheet 2,** Data of biofilm weight. **Sheet 3**, Bacterial cell numbers data of biofilm communities based on qPCR. **Sheet 4**, Metabolites exchange profile analyzed by SEMTANA. **Sheet 5**, M9 minimal medium composition and global metabolic interaction potential (MRO) value. **Sheet 6**, Data of phenotype microarray assay.

**Supplementary Table 3.** Tables containing the data used in the supplementary figures. **Sheet 1**, Summary table of bacterial relative abundance at genus or higher taxonomy level in the soil biofilm and solution. **Sheet 2**, Growth curve data in TSB medium. **Sheet 3 – 7**, Growth curve data in M9 glucose medium. **Sheet 8**, Endpoint OD_600_ data of metabolite exchange validation. **Sheet 9.** Growth curve data in Chr supernatant
